# Supramolecular linker-directed assembly of a ‘trap-and-diffusion’ MOF for one-step purification of polymer-grade C_2_H_4_ from C_2_ hydrocarbons

**DOI:** 10.1093/nsr/nwaf548

**Published:** 2025-12-01

**Authors:** Jia-Peng Han, Han Fang, Hongliang Huang, Zheng-Yu Su, Haichao Wang, Bo Zhang, Michael J Zaworotko, Shi-Qiang Wang, Mei-Hui Yu, Ze Chang, Xian-He Bu

**Affiliations:** School of Materials Science and Engineering, TKL of Metal and Molecule-Based Material Chemistry, Nankai University, Tianjin 300350, China; School of Materials Science and Engineering, TKL of Metal and Molecule-Based Material Chemistry, Nankai University, Tianjin 300350, China; State Key Laboratory of Advanced Separation Membrane Materials, School of Chemical Engineering and Technology, Tiangong University, Tianjin 300387, China; School of Materials Science and Engineering, TKL of Metal and Molecule-Based Material Chemistry, Nankai University, Tianjin 300350, China; School of Materials Science and Engineering, TKL of Metal and Molecule-Based Material Chemistry, Nankai University, Tianjin 300350, China; School of Materials Science and Engineering, TKL of Metal and Molecule-Based Material Chemistry, Nankai University, Tianjin 300350, China; Department of Chemical Sciences and Bernal Institute, University of Limerick, Limerick V94 T9PX, Ireland; Department of Chemical Sciences and Bernal Institute, University of Limerick, Limerick V94 T9PX, Ireland; School of Materials Science and Engineering, TKL of Metal and Molecule-Based Material Chemistry, Nankai University, Tianjin 300350, China; School of Materials Science and Engineering, TKL of Metal and Molecule-Based Material Chemistry, Nankai University, Tianjin 300350, China; School of Materials Science and Engineering, TKL of Metal and Molecule-Based Material Chemistry, Nankai University, Tianjin 300350, China; State Key Laboratory of Elemento-Organic Chemistry, College of Chemistry, Nankai University, Tianjin 300071, China

**Keywords:** metal–organic frameworks, supramolecular linker, molecular pockets, ethylene purification, hydrocarbon separation

## Abstract

Simultaneously removing ethane (C_2_H_6_) and acetylene (C_2_H_2_) from ethylene (C_2_H_4_) streams is advantageous for industrial production yet remains challenging for physisorbents. Herein, we report a microporous metal–organic framework (MOF), **NKM-47**, that achieves one-step C_2_H_4_ purification. **NKM-47** was designed via a supramolecular linker-directed assembly approach and features a ‘trap-and-diffusion’ porous architecture composed of orthogonally arranged molecular pockets and 1D channels. The N/O-rich molecular pockets selectively capture the smallest and largest C_2_ species (C_2_H_2_ and C_2_H_6_) while the channels permit preferential diffusion of medium-sized C_2_H_4_. **NKM-47** enables one-step production of polymer-grade C_2_H_4_ (99.99% purity) from both binary and ternary C_2_ gas mixtures under ambient conditions. This study presents the first example of a trap-and-diffusion mechanism for C_2_ hydrocarbon separation in MOFs, enabling the single-step purification of C_2_H_4_ through the selective diffusion of a species with intermediate physicochemical properties.

## Teaser

This work constructed a "trap-and-diffusion" MOF via a supramolecular linker directed assembly strategy specifically tailored to address the formidable challenge of one-step C_2_H_4_ purification from complex C_2_ mixtures, achieving simultaneous, selective exclusion of both C_2_H_2_ and C_2_H_6_ impurities.

## INTRODUCTION

Ethylene (C_2_H_4_), the highest-volume product of the petrochemical industry, serves as a key feedstock for the manufacture of a wide range of chemicals and polymers [1–3]. However, the current industrial process for ethylene production via hydrocarbon steam cracking inevitably generates impurities, including ethane (C_2_H_6_) and acetylene (C_2_H_2_) [4]. The presence of these byproducts poses a significant challenge, as polymer-grade C_2_H_4_ requires the efficient removal of C_2_H_6_ and C_2_H_2_ from C_2_ hydrocarbon mixtures [5,6]. Unfortunately, separating these three gases remains difficult due to their similar molecular sizes and physicochemical properties [7–9]. Conventional purification techniques, such as catalytic hydrogenation, solvent extraction and cryogenic distillation, are energy-intensive and involve complex engineering processes [10,11]. In contrast, adsorptive separation, known for its high energy efficiency and operational simplicity, represents a promising alternative [12]. Among these methods, the direct purification of C_2_H_4_ streams without an additional desorption step is particularly attractive, as it can significantly reduce the energy footprint [13,14]. In this regard, metal–organic frameworks (MOFs) have emerged as benchmark sorbents thanks to their amenability to design and tunable composition and pore structure [15–18].

Unlike MOFs designed for C_2_H_4_-selective adsorption, the development of MOF adsorbents that preferentially capture C_2_H_6_ and C_2_H_2_ over C_2_H_4_ remains limited and challenging, owing to the intermediate molecular size, quadrupole moment and polarizability of C_2_H_4_ (Table S1) [19–22]. Thus far, only a few MOFs—notably NPU-1, NPU-2 and NPU-3—have achieved the single-step purification of C_2_H_4_ from ternary C_2_ hydrocarbon mixtures [23]. In general, introducing electronegative heteroatoms (e.g. nitrogen or fluorine) can establish strong Lewis acid–base interactions between the basic heteroatoms and the acidic hydrogen atoms of C_2_H_2_ [24,25]. Meanwhile, because C_2_H_6_ has greater polarizability, larger kinetic diameter and van der Waals (vdW) surface area and more C–H bonds than C_2_H_4_, MOFs with nonpolar/inert pore surfaces that incorporate aromatic or aliphatic groups for supramolecular interactions may enhance C_2_H_6_ selectivity [26–28]. For example, Li *et al.* reported a MOF adsorbent, JNU-2, featuring carboxylate oxygen-decorated nonpolar pores, which enables the selective separation of C_2_H_4_ from C_2_H_6_/C_2_H_4_/C_2_H_2_ mixtures [29]. The synergy between tailored pore dimensions and functional groups can enhance supramolecular interactions with C_2_H_2_ and C_2_H_6_ over C_2_H_4_, as demonstrated in CuIn(ina)_4_ [30], CAU-10-X [31], CuTiF_6_-TPPY [7], UiO-67-(NH_2_)_2_ [32], Al-PyDC [22], LUDLAZ [33], A-66 [34] and so on. Although this surface-engineering approach has proven effective and prompted the development of related MOF adsorbents, further design of the pore architecture remains unfulfilled. Notably, Li and Lu *et al.* demonstrated that an orthogonal array of binding sites along diffusion channels in a MOF enables high-purity propylene production from propane/propylene mixtures and ethylene purification from C_2_–C_4_ alkynes [35,36]. This suggests that a more delicate C_2_H_6_/C_2_H_2_-selective porous system could be developed by integrating the aforementioned pore surface-engineering approach with an orthogonal-array pore architecture in MOFs.

Nevertheless, rational design of such a ‘trap-and-diffusion’ porous framework remains underexplored due to the structural complexity of such heterogeneous pore architectures. Self-aggregation of organic linkers, i.e. pure-supramolecular-linkers (PSLs), has recently been proposed as an approach to construct unique MOF architectures [22,37–39]. For example, NJU-Bai52 and NJU-Bai53 comprised eight-connected PSLs with four-connected M_3_O clusters, forming structures with 1D hourglass-shaped channels and octahedral cages [37]. Subsequently, semirigid amide-functionalized linker dimers were employed as PSLs to construct rod In-MOFs, generating a complex network with two types of cage-like pore geometries [40]. Generally, the utilization of PSLs could introduce steric hindrance and geometric constraints during coordination assembly, leading to geometry mismatches that drive the formation of heterogeneous pore architectures in MOFs, which in turn can enhance the gas-separation performance of the material [41,42].

**Scheme 1.**
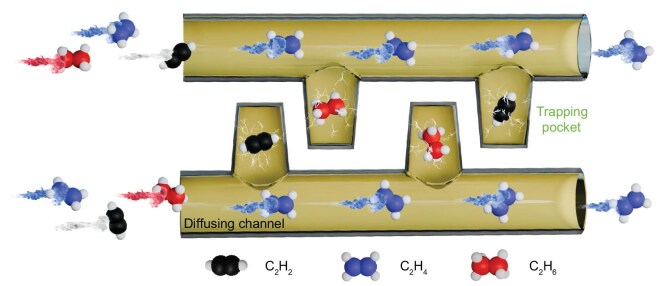
Schematic illustration of the ‘trap-and-diffusion’ porous framework of **NKM-47**, which features orthogonally arranged cupola-shaped molecular pockets and 1D channels. Preferential capture of C_2_H_6_ and C_2_H_2_ within the pockets facilitates the diffusion and separation of C_2_H_4_.

Building on these considerations, we introduced a tetratopic linker, 5,5′-di(1H-1,2,4-triazol-1-yl)-[1,1′-biphenyl]-3,3′-dicarboxylic acid (H_2_DTBDC), for the construction of porous MOFs. The H_2_DTBDC linker was chosen because of two key features: (i) its highly conjugated, planar geometry helps stabilize aggregates through π–π stacking, which may exhibit multiple supramolecular conformers and facilitate the assembly of heterogeneous pore architectures; (ii) its aromatic groups and electronegative nitrogen atoms may enhance the C_2_H_4_ purification ability from ternary C_2_ mixtures. A survey of H_2_DTBDC connected to any metal using the Cambridge Structural Database (CSD version 5.46, November 2024) revealed that this linker was first utilized in coordination chemistry (Fig. S1). In this contribution, we present the construction of a heterogeneous pore architecture within a new porous MOF, [Zn_2_(DTBDC)_2_]*_n_*·*x*(solvents) (**NKM-47**, NKM = Nankai materials). This hierarchical design comprises orthogonally arranged cupola-shaped pockets that selectively trap C_2_H_6_ and C_2_H_2_, while providing diffusion pathways for the preferential diffusion of C_2_H_4_, thereby enabling the effective one-step purification of C_2_H_4_ from ternary C_2_ hydrocarbon mixtures (Scheme 1).

## RESULTS AND DISCUSSION

**Figure 1.**
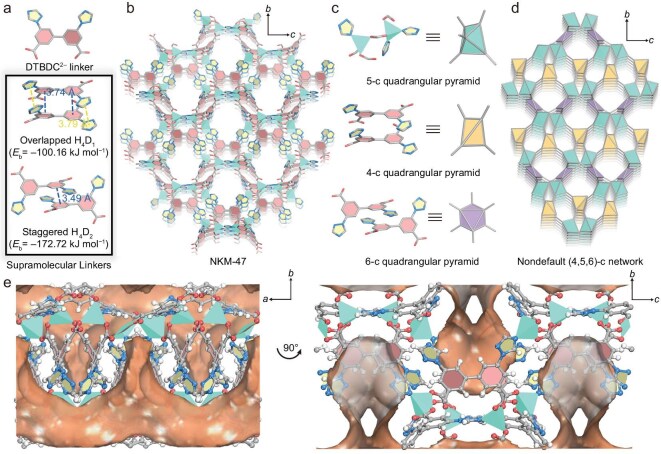
(a) DTBDC^2−^ linker, overlapped D_1_^4−^ and staggered D_2_^4−^ dimers formed through its stacking interactions. (b) Structural representation of **NKM-47**. (c) Molecular building blocks and corresponding simplified SBUs in **NKM-47**. (d) Simplified (4,5,6)-c topology network of **NKM-47**. (e) Connolly surface representation of **NKM-47** presents orthogonally arranged molecular pockets and channels along the *c*-axis and *a*-axis, respectively.

The solvothermal reaction of Zn(NO_3_)_2_·6H_2_O and H_2_DTBDC in the mixture of *N,N*-dimethylformamide (DMF) and tetrafluoroboric acid yielded transparent block crystals of **NKM-47**, formulated as [Zn_2_(DTBDC)_2_]*_n_*·*x*(solvents). Single-crystal X-ray diffraction analysis revealed that **NKM-47** crystallizes in the monoclinic space group *C*2/*c* (Table S2). Inspection of the asymmetric unit revealed two crystallographically independent four-connected Zn(II) ions: one coordinated by three carboxylate groups and one triazole group and the other by two carboxylate and two triazole groups (Fig. S3). The resulting binuclear zinc cluster, [Zn_2_(O_2_C–)_4_(N_3_C_2_–)_3_], is stabilized by a μ-carboxylate bridge between two types of adjacent Zn(II) ions, featuring *C*_1_ symmetry. As anticipated, two PSLs were formed through dimerization of the DTBDC^2−^ linkers. Mediated by intermolecular aromatic π–π stacking, two crystallographically independent DTBDC^2−^ linkers pair with their symmetry-equivalent counterparts to create two new supramolecular linkers: an overlapped 4-c H_4_D_1_ with *C*_2_ symmetry and a staggered 6-c H_4_D_2_ with *C_i_* symmetry (Fig. 1a). Based on a system energy calculation by using density functional theory (DFT) optimization through the Dmol^3^ module [43], both dimers demonstrate favorable energetics, with binding energies of −100.16 and −172.72 kJ mol^−1^, respectively. These relatively high binding energies indicate that the PSLs are sufficiently stable to drive the direct assembly of the resulting framework. By virtue of this PSL strategy, the binuclear zinc clusters are cross-linked by deprotonated D_1_^4−^ and D_2_^4−^ linkers to form an intricate 3D network. The obtained **NKM-47** framework features aligned hexagonal channels with an aperture of ∼6.7 Å along the *a*-axis (Fig. 1b). Interestingly, cupola-shaped pockets enclosed by four D_1_^4−^ linker sidewalls and one D_2_^4−^ linker subface are aligned in an alternating up–down manner along the channels, providing access to the channel with an exposed aperture of ∼5.8 Å along the *c*-axis (Fig. 1e and Fig. S2). The pocket size is slightly larger than that of a single C_2_ light hydrocarbon molecule, allowing confinement effects and multiple host–guest interactions. Meanwhile, the Zeo++ code was utilized to analyse the pore architecture of **NKM-47**, determining its pore-limiting diameter as 6.59 Å and its largest cavity diameter at 7.91 Å [44]. The solvent-accessible free volume of **NKM-47** was calculated to be 46.2% with a probe radius of 1.2 Å, as determined by using PLATON software [45].

Topologically, **NKM-47** exhibits a rare (4,5,6)-c network arising from the coexistence of two kinds of supramolecular linkers (Fig. 1c and Fig. S4). The binuclear zinc cluster of [Zn_2_(O_2_C–)_4_(N_3_C_2_–)_3_] in **NKM-47** can be described as a distorted 5-c quadrangular pyramid secondary building unit (SBU), while *C_i_* symmetric D_1_^4−^ and *C*_2_ symmetric D_2_^4−^ can be regarded as distorted 4-c tetrahedral and 6-c octahedral SBUs, respectively. This geometric mismatch yields an intricate net with the Schläfli symbol (4^3^.6^3^)(4^6^.6^4^)_2_(4^6^.6^7^.8^2^) (Fig. 1d) [46,47].

**Figure 2.**
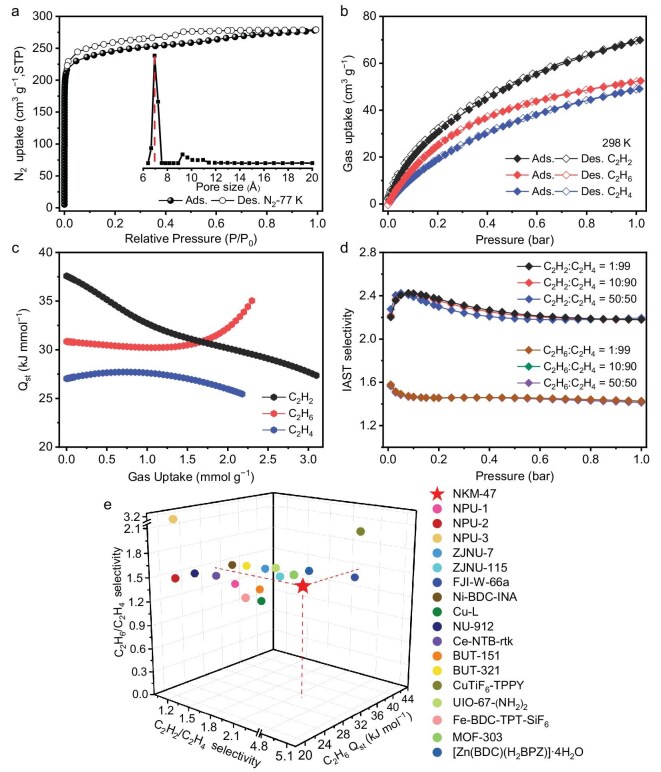
(a) N_2_ sorption isotherm of **NKM-47** at 77 K with the inset of the pore-size distribution. (b) C_2_H_2_, C_2_H_4_, and C_2_H_6_ sorption isotherms of **NKM-47** at 298 K. (c) Isosteric heats of adsorption (*Q*_st_) of C_2_H_2_, C_2_H_4_ and C_2_H_6_. (d) IAST selectivity for 1:99, 10:90 and 50:50 (v:v) C_2_H_2_/C_2_H_4_ and C_2_H_6_/C_2_H_4_ at 298 K. (e) Comprehensive comparison of **NKM-47** with relevant MOFs capable of achieving one-step C_2_H_4_ purification under ambient conditions, focusing on the selectivity for C_2_H_2_/C_2_H_4_ and C_2_H_6_/C_2_H_4_ mixtures and the *Q*_st_ of C_2_H_6_.

A bulk crystal sample of **NKM-47** exhibited high phase purity, as evidenced by the well-matched calculated and experimental powder X-ray diffraction (PXRD) patterns (Fig. S5). Furthermore, the PXRD patterns remained unchanged after thermal activation and solvent exchange, indicating the rigid nature of **NKM-47** (Figs S5 and S6). Variable-temperature PXRD analysis demonstrated that **NKM-47** preserved crystallinity up to 340°C (Fig. S7). Thermogravimetric analysis indicated that **NKM-47** was stable up to 400°C (Fig. S8). To evaluate the porosity, freshly prepared samples were solvent-exchanged with acetone for 3 days and then degassed under a high vacuum at 80°C for 12 h before the gas sorption measurements. As shown in Fig. 2a, the N_2_ sorption isotherm of **NKM-47** exhibits a steep uptake at low pressure, characteristic of micropore filling, followed by a distinct hysteresis loop at higher pressures, with a Brunauer–Emmett–Teller surface area of 961.43 m^2^ g^−1^ (Fig. S9). To probe the origin of hysteresis, *in situ* N_2_-dosed PXRD measurements were conducted to monitor potential structural changes during the sorption process. The diffraction peaks of **NKM-47** show negligible shifts during the test (Fig. S10), confirming the rigidity of the framework and ruling out structural flexibility as the cause of hysteresis. Instead, the observed hysteresis arises from the unique pore architecture: the orthogonal pockets are accessible exclusively through narrow channels. This sorption behavior corresponds to the type H4 defined by the International Union of Pure and Applied Chemistry (IUPAC), where desorption is kinetically hindered by channel blocking [29,48,49]. The experimental total pore volume derived from the N_2_ isotherm is 0.43 cm^3^ g^−1^, in agreement with the theoretical pore volume of 0.43 cm^3^ g^−1^. The pore-size distribution estimated by using the quenched solid DFT model ranges from 6.4 to 7.5 Å, with a width centered on 6.9 Å, consistent with the crystallographic data [50,51].

The orthogonal-array microporous architecture of **NKM-47**, characterized by an N/O-rich pore environment, prompted us to investigate its separation performance for C_2_ hydrocarbons [26–28]. Single-component sorption isotherms for C_2_H_2_, C_2_H_4_, and C_2_H_6_ were measured at 273 and 298 K up to a pressure of 1 bar. The adsorption capacities at 298 K and 1 bar were 69.90 cm^3^ g^−1^ for C_2_H_2_, 49.11 cm^3^ g^−1^ for C_2_H_4_ and 52.53 cm^3^ g^−1^ for C_2_H_6_, respectively (Fig. 2b). The capacities at 273 K increased to 91.60 cm^3^ g^−1^ for C_2_H_2_, 65.36 cm^3^ g^−1^ for C_2_H_4_ and 83.89 cm^3^ g^−1^ for C_2_H_6_ (Fig. S11). At both temperatures, **NKM-47** exhibited higher uptakes for C_2_H_2_ and C_2_H_6_ than that for C_2_H_4_ across the full pressure range, following the affinity order C_2_H_2_ > C_2_H_6_ > C_2_H_4_. This trend suggests that **NKM-47** has the potential to purify C_2_H_4_ from ternary C_2_ hydrocarbons in a single step.

The interaction strengths between **NKM-47** and the three C_2_ gases' molecules were evaluated based on their isosteric heats of adsorption (*Q*_st_), obtained through fitting variable-temperature single-component adsorption isotherms with the dual-site Langmuir equation (Table S3 and Figs S12–S14). The zero-coverage *Q*_st_ values for C_2_H_2_ (37.58 kJ mol^−1^) and C_2_H_6_ (30.84 kJ mol^−1^) were found to be higher than that of C_2_H_4_ (27.03 kJ mol^−1^), consistently with the observed uptake trend (Fig. 2c). The *Q*_st_ for C_2_H_6_ is significantly lower than that for most reported C_2_H_6_-selective MOFs, e.g. FJI-W-66a (40.48 kJ mol^−1^) [52], CuTiF_6_-TPPY (34.2 kJ mol^−1^) [7] and BUT-151 (31.06 kJ mol^−1^) [53], and only slightly higher than that for NPU-1 (29.10 kJ mol^−1^) [23], ZJNU-7 (29.7 kJ mol^−1^) [54], Ni-BDC-INA (29.01 kJ mol^−1^) [55], Cu-L (28.4 kJ mol^−1^) [56] and UiO-67-(NH_2_)_2_ (26.5 kJ mol^−1^) [32], implying that desorption and regeneration would require modest energy (Fig. 2e). Meanwhile, the *Q*_st_ for C_2_H_2_ is much lower than that observed for FJI-W-66a (42.36 kJ mol^−1^) [52] and Zr-TCA (43.8 kJ mol^−1^) [57]. These moderate *Q*_st_ values provide strong yet manageable affinities for C_2_H_6_ and C_2_H_2_ capture, enabling facile adsorbent regeneration with a minimal energy penalty.

Inspired by the selective adsorption of C_2_H_2_ and C_2_H_6_ over C_2_H_4_ by **NKM-47**, ideal adsorbed solution theory (IAST) was applied to quantify the adsorption selectivities of **NKM-47** for both C_2_H_2_/C_2_H_4_ and C_2_H_6_/C_2_H_4_ mixtures with different ratios, i.e. 1:99, 10:90 and 50:50 (v:v), at 298 K, as fitting via the dual-site Langmuir–Freundlich equation (Table S4 and Fig. S15). As shown in Fig. 2d, the IAST selectivities of C_2_H_6_/C_2_H_4_ mixtures for 1:99, 10:90 and 50:50 ratios can reach 1.43, 1.43 and 1.41 at 1 bar, respectively. These values outperform those of several well-known MOFs for equimolar C_2_H_6_/C_2_H_4_, e.g. NPU-1 (1.32) [23], FJI-W-66a (1.4) [52], Fe-BDC-TPT-SiF_6_ (1.4) [58], Cu-L (1.2) [56] and BUT-151 (1.26) [53], but are lower than those of the top-performing porous adsorbents for this application, e.g. CuTiF_6_-TPPY (2.12) [7], NPU-3 (3.21) [23], UiO-67-(NH_2_)_2_ (1.7) [32] and BUT-321 (1.6) [59]. The IAST selectivities for the C_2_H_2_/C_2_H_4_ mixtures (1:99, 10:90 and 50:50) at 298 K and 1 bar for **NKM-47** are 2.18, 2.18 and 2.19, respectively. The equimolar C_2_H_2_/C_2_H_4_ selectivity outperforms those of most MOFs for one-step C_2_H_4_ purification, e.g. NPU-1 (1.4) [23], NPU-2 (1.25) [23], NPU-3 (1.32) [23], ZJNU-7 (1.77) [54], Ni-BDC-INA (1.37) [55], Cu-L (1.8) [56], NU-912 (1.2) [60], Ce-NTB-rtk (1.36) [61], ZJNU-115 (2.05) [62], [Zn(BDC)(H_2_BPZ)]·4H_2_O (1.6) [3], BUT-151 (1.61) [53], BUT-321(1.6) [59], Fe-BDC-TPT-SiF_6_ (2.0) [58] and UiO-67-(NH_2_)_2_ (2.1) [32], but is lower than those of top-performing MOFs, i.e. MOF-303 (2.4) [63], FJI-W-66a (2.31) [52] and CuTiF_6_-TPPY (5.03) [7]. Simultaneously, to better understand the separation efficiency of **NKM-47**, the separation potential (*Δq*, a comprehensive indicator of adsorption capacity and selectivity) was calculated, allowing further evaluation of the maximum recoverable productivity of pure components by the MOF adsorbents in fixed beds [64,65]. At 298 K and 1 bar, for the 1/99 and 50/50 C_2_H_2_/C_2_H_4_ mixture, the *Δq* values were calculated to be 2.526 and 0.999 mmol g^−1^, respectively (Figs S16 and S17). For the C_2_H_6_/C_2_H_4_ mixture, the maximum productivity of C_2_H_4_ recovery by **NKM-47** reached 0.809 and 0.389 mmol g^−1^ for the 10/90 and 50/50 C_2_H_6_/C_2_H_4_ mixtures, respectively. These results collectively support the potential of **NKM-47** for purifying C_2_H_4_ from C_2_H_2_/C_2_H_4_ and C_2_H_6_/C_2_H_4_ mixtures under ambient conditions.

**Figure 3.**
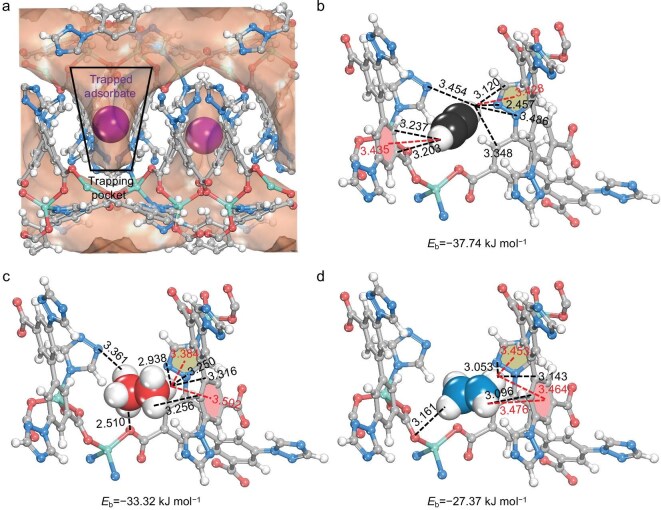
(a) Connolly surface of **NKM-47**, demonstrating the arrangement of molecular trapping pockets. Calculated adsorption regions and specific interaction between gas molecules and the pocket for (b) C_2_H_2_, (c) C_2_H_6_, and (d) C_2_H_4_.

To further confirm the potential of C_2_ separation of **NKM-47**, adsorption sites and interaction discrepancies were calculated based on first-principles dispersion-corrected DFT. The Connolly surface of the crystal structures clearly shows the orthogonal-array molecular pockets of **NKM-47**, which was further proved to be the primary adsorption regions of all C_2_ hydrocarbon molecules (Fig. 3a). Each confined pocket can accommodate one gas molecule, with all three kinds of C_2_ molecules binding to the pocket through multiple synergistic hydrogen-bonding interactions with the DTBDC^2−^ linkers. Compared with C_2_H_4_, C_2_H_2_ and C_2_H_6_ exhibit more extensive interactions and stronger binding energies, indicating preferential binding of C_2_H_6_ and C_2_H_2_ over C_2_H_4_ within the pocket. The calculated static binding energies are −37.74 kJ mol^−1^ for C_2_H_2_ and −33.32 kJ mol^−1^ for C_2_H_6_—both stronger than that of C_2_H_4_ (−27.37 kJ mol^−1^) and in agreement with the calculated *Q*_st_ values. A more detailed analysis of the simulated crystal structures reveals that C_2_ hydrocarbons engage in synergetic host–guest interactions concentrated in three key regions within the pocket: the benzene ring region, the triazole heterocycle region, and the carboxylate region. As shown in Fig. 3b, the H atoms of C_2_H_2_ form two C−H···π interactions with a benzene ring and a triazole ring of the DTBDC^2−^ linkers, exhibiting H···π distances of 3.435 and 3.428 Å, respectively. In addition, the C_2_H_2_ molecule is stabilized by two aromatic rings and three triazole rings of the surrounding DTBDC^2−^ linkers via four C–H···C (ranging from 3.120 and 3.348 Å) and three C–H···N (ranging from 2.457 to 3.486 Å) H-bonding interactions. The C_2_H_6_ molecule reveals a similar interaction with the confined pocket to that of C_2_H_4_ (Fig. 3c). The C_2_H_6_ molecule is bonded via two C–H···C (3.256 and 3.316 Å) and three C–H···N (ranging from 2.938 to 3.361 Å) hydrogen bonds to the surrounding carbon and nitrogen atoms. Notably, the C_2_H_6_ molecule interacts with the oxygen atom of the carboxylate group to form C–H···O hydrogen bonds, whose length is 2.510 Å. Simultaneously, it also engages in two C–H···π interactions (3.384 and 3.502 Å) with a triazole ring and a benzene ring, further enhancing its interactions with the pocket. In contrast, the interactions between the planar C_2_H_4_ molecule and the pocket surface are weaker than those of both C_2_H_2_ and C_2_H_6_, demonstrating lower affinity with the inner channel walls. The C_2_H_4_ molecule shows multiple supramolecular interactions through two C–H···C bonds (3.096 and 3.143 Å), one C–H···N bond (3.053 Å), one C–H···O bond (3.161 Å) and three C–H···π vdW interactions (ranging from 3.453 to 3.476 Å) with the framework (Fig. 3d). The weaker interactions between the C_2_H_4_ molecule and the pocket make it less competitive for adsorption than C_2_H_2_ and C_2_H_6_. Consequently, the C_2_H_2_ and C_2_H_6_ molecules preferentially occupy the trapping pockets. These findings are consistent with the experimental data discussed above, further validating the potential of **NKM-47** for single-step purification of C_2_H_4_ from ternary C_2_ light hydrocarbon mixtures.

**Figure 4.**
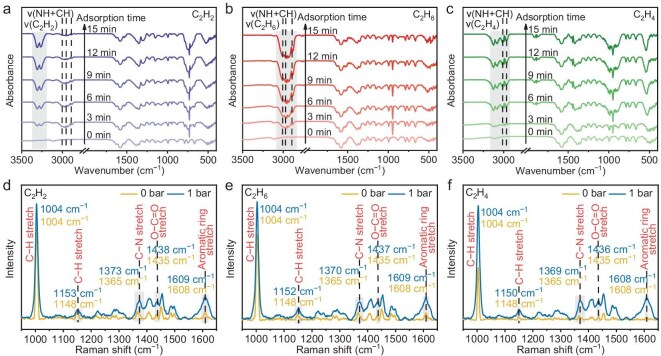
*In situ* FTIR spectra of the activated **NKM-47** sample exposed to (a) C_2_H_2_, (b) C_2_H_6_ and (c) C_2_H_4_ gas at room temperature. *In situ* Raman spectra of the activated **NKM-47** sample exposed to (d) C_2_H_2_, (e) C_2_H_6_ and (f) C_2_H_4_ gas at room temperature.

To investigate the impact of the abundant binding sites on the **NKM-47** surface on the adsorption of different C_2_ gases in more detail, we performed *in situ* Fourier transform infrared spectroscopy (FTIR) to elucidate the mechanism of the host–guest interactions. When C_2_H_2_, C_2_H_6_, or C_2_H_4_ was loaded into activated **NKM-47**, distinct phenomena were observed: prominent stretching vibration peaks corresponding to each gas appeared in the spectra (Fig. 4a–c and Fig. S18). Specifically, multiple absorption peaks at 3358–3192 cm^−1^ were attributed to ν(C_2_H_2_), the peaks within 3086–2838 cm^−1^ to ν(C_2_H_6_) and the peaks within 3165–2925 cm^−1^ to ν(C_2_H_4_) [7,63]. Concurrently, characteristic ν(NH + CH) peaks emerged in the spectra after gas loading. For C_2_H_2_ adsorption, ν(NH + CH) bands appeared at 3004, 2958 and 2891 cm^−1^; for C_2_H_6_ adsorption, they appeared at 3003, 2961 and 2890 cm^−1^; and for C_2_H_4_ adsorption, they appeared at 3004 and 2890 cm^−1^, indicating interactions between the triazolyl groups/aromatic rings and the gases [63,66,67]. Notably, during the C_2_H_2_ and C_2_H_6_ adsorption, distinct characteristic absorption bands were observed within 3 min, intensifying significantly over time with more pronounced changes in the ν(NH + CH) bands. These results demonstrate that **NKM-47** adsorbs C_2_H_2_ and C_2_H_6_ more strongly and rapidly than does C_2_H_4_.

Subsequently, supplementary *in situ* Raman spectroscopy was employed to further elucidate the role of N/O sites in the adsorption of C_2_ gases. Under a vacuum, Raman characteristic peaks of the triazolyl groups included a C–H stretch at 1004 cm^−1^ and a C–N stretch at 1365 cm^−1^, while the carboxylate group exhibited an O–C=O stretch at 1435 cm^−1^ (Fig. 4d–f) [68,69]. Peaks at 1148 and 1608 cm^−1^ were attributed to an aromatic C–H stretch and ring stretch, respectively [70,71]. When the pressure reached 1 bar upon introducing each C_2_ gas, the Raman peak intensity of the framework groups was significantly enhanced. Moreover, partial peaks corresponding to triazolyl, carboxylate and aromatic ring groups exhibited noticeable shifts after gas introduction. Experimental results combined with theoretical calculations collectively demonstrate the contributions of triazolyl, carboxylate and aromatic ring groups to gas adsorption. Compared with C_2_H_4_, the introduction of C_2_H_2_ and C_2_H_6_ induced more pronounced spectral shifts, indicating enhanced adsorption interactions with these gases.

**Figure 5.**
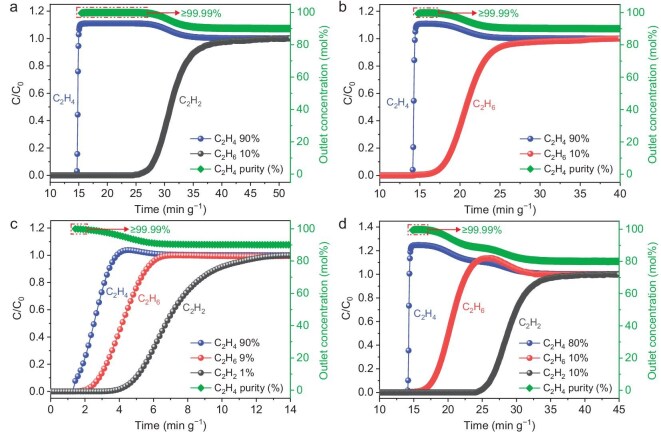
Experimental column breakthrough curves of **NKM-47** for (a) 10/90 C_2_H_2_/C_2_H_4_ and (b) 10/90 C_2_H_6_/C_2_H_4_ binary mixtures, conducted at 298 K and 1 bar with a gas-flow rate of 4.0 mL min^−1^. (c) Experimental column breakthrough curves of **NKM-47** for 1/9/90 C_2_H_2_/C_2_H_6_/C_2_H_4_ ternary mixtures, conducted at 298 K and 1 bar with a gas-flow rate of 20.0 mL min^−1^. (d) Experimental column breakthrough curves of **NKM-47** for 10/10/80 C_2_H_2_/C_2_H_6_/C_2_H_4_ ternary mixtures, conducted at 298 K and 1 bar with a gas-flow rate of 4.0 mL min^−1^.

Motivated by the dual features of high selectivity for C_2_H_2_/C_2_H_4_ and reversed selectivity for C_2_H_6_/C_2_H_4_, the dynamic separation performances of **NKM-47** for the binary 10/90 (v/v) C_2_H_2_/C_2_H_4_ and C_2_H_6_/C_2_H_4_ mixtures were evaluated through breakthrough experiments at 298 K. Feed gases were passed through an **NKM-47-**packed column at a flow rate of 4 mL min^−1^. As shown in Fig. 5a, the breakthrough curve for the C_2_H_2_/C_2_H_4_ (10/90, v/v) mixture indicates that C_2_H_4_ eluted first from the adsorption bed at 14.75 min g^−1^, followed by C_2_H_2_ at a breakthrough time of 27.31 min g^−1^, resulting in a breakthrough interval of 12.56 min g^−1^. Likewise, the breakthrough curves in Fig. 5b demonstrate that C_2_H_4_ eluted preferentially before C_2_H_6_, with a breakthrough interval of 3.36 min g^−1^. During the time interval of breakthrough for the C_2_H_2_/C_2_H_4_ and C_2_H_6_/C_2_H_4_ gas mixtures, high-purity C_2_H_4_ was directly obtained from the outlet (Figs S19 and S20). The dynamic mixture separation results confirm that **NKM-47** exhibits greater affinity for C_2_H_2_ and C_2_H_6_ than for C_2_H_4_, aligning well with the previously discussed adsorption behavior. These breakthrough results suggest the potential for one-step C_2_H_2_ purification from ternary C_2_ hydrocarbon mixtures.

The separation performance of **NKM-47** for ternary C_2_H_2_/C_2_H_6_/C_2_H_4_ (1/9/90 and 10/10/80, v/v/v) mixtures at 1 bar and 298 K was investigated. A higher gas-flow rate of 20.0 mL min^−1^ was employed for the 1/9/90 ternary gas mixtures. The selective extraction of C_2_H_4_ from the ternary C_2_ gas mixtures was indeed effectively realized by passing these mixtures through a packed column containing activated **NKM-47**. As illustrated in Fig. 5c, in the breakthrough curve of the 1/9/90 C_2_H_2_/C_2_H_6_/C_2_H_4_ mixture, C_2_H_4_ broke through first at 1.44 min g^−1^, without C_2_H_2_ or C_2_H_6_ being detected before their breakthrough. Subsequently, C_2_H_6_ was obtained at 2.51 min g^−1^, while C_2_H_2_ had the longest retention time, recorded at 4.67 min g^−1^, which aligns with the observed relative affinity for C_2_ hydrocarbons. Given the need to evaluate the separation capability of **NKM-47** through different concentrations of C_2_ gas mixtures, the separation of 10/10/80 C_2_H_2_/C_2_H_6_/C_2_H_4_ mixture was studied at a gas-flow rate of 4.0 mL min^−1^ (Fig. 5d). The test revealed that C_2_H_4_ eluted first from the adsorption bed at 14.18 min g^−1^, followed by C_2_H_6_ at 17.16 min g^−1^, while C_2_H_2_ exhibited the longest retention time of 25.47 min g^−1^. During the breakthrough between C_2_H_4_ and C_2_H_6_, analysis revealed the presence of high-purity C_2_H_4_ within a single step (Figs S21 and S22). These adsorbate-dependent breakthrough behaviors demonstrate the preferential binding of C_2_H_6_/C_2_H_2_ and the diffusion of C_2_H_4_, marking the first experimental validation of a ‘trap-and-diffusion’ system in MOF that enables one-step C_2_H_4_ purification with >99.99% purity. To evaluate the recyclability and reusability of **NKM-47**, which is essential for practical applications, gas purging was employed for regeneration, followed by three consecutive breakthrough experiments with the ternary C_2_ mixtures. As shown in Figs S23 and S24, the time intervals between the breakthroughs of C_2_H_4_ and C_2_H_2_/C_2_H_6_ remained consistent across the three regeneration cycles. Considering its high adsorption capacities for C_2_H_2_ and C_2_H_6_, reversed C_2_H_6_/C_2_H_4_ selectivity and outstanding separation performance for binary and ternary C_2_ mixtures, **NKM-47** holds excellent promise as a material for the one-step purification of C_2_H_4_.

## CONCLUSION

We report a PSL strategy utilizing the previously unexplored H_2_DTBDC linker to prepare **NKM-47**—a MOF characterized by orthogonally arranged molecular pockets and 1D channels. These trapping pockets, functionalized with synergistic benzene and triazole moieties, endow **NKM-47** with strong adsorption selectivity for both C_2_H_6_ and C_2_H_2_ over C_2_H_4_. Dynamic breakthrough experiments conducted with binary and ternary C_2_ hydrocarbon mixtures validated the ability of **NKM-47** to achieve the one-step production of high-purity C_2_H_4_ thanks to its pocket-and-channel pore structure. This work represents a rare case of a trap-and-diffusion mechanism that fulfills the selective diffusion of molecules with intermediate physicochemical properties, addressing the long-standing challenge of one-step C_2_H_4_ separation. Therefore, **NKM-47** has emerged as a promising candidate for sustainable and economically viable C_2_H_4_ purification, representing a design strategy that can afford MOFs optimized for high-performance separation.

## EXPERIMENTAL SECTION

The experimental details are listed in the Supporting Information.

## Supplementary Material

nwaf548_Supplemental_Files

## Data Availability

The data that support the findings of this study are available from the corresponding author upon reasonable request.
