# Supplementary material for: Supramolecular linker-directed assembly of a ‘trap-and-diffusion’ MOF for one-step purification of polymer-grade C2H4 from C2 hydrocarbons
Source: Natl Sci Rev. 2025 Dec 1;13(3):nwaf548. doi: 10.1093/nsr/nwaf548 (PMC12887298; doi:10.1093/nsr/nwaf548)
Supplement: nwaf548_Supplemental_Files [file nwaf548_supplemental_files.zip › NSR-SI_Revision.pdf]

Supporting Information

Supramolecular Linker Directed Assembly of a "Trap-and-Diffusion" MOF for One-Step Purification of Polymer-Grade C_2H_4 from C2 Hydrocarbons

Jia-Peng Han[1#], Han Fang[1#], Hongliang Huang[3], Zheng-Yu Su[1], Haichao Wang[1], Bo Zhang[1], Michael J. Zaworotko[4], Shi-Qiang Wang[4], Mei-Hui Yu[1]*, Ze Chang[1]*, and Xian-He Bu[1,2]

[1]*School of Materials Science and Engineering, TKL of Metal and Molecule-Based Material Chemistry, Nankai University, Tianjin 300350, China.*

[2]*State Key Laboratory of Elemento-Organic Chemistry, College of Chemistry, Nankai University, Tianjin 300071, China.*

[3]*State Key Laboratory of Advanced Separation Membrane Materials, Tiangong University, Tianjin 300387, China.*

[4]*Department of Chemical Sciences and Bernal Institute, University of Limerick, Limerick, Ireland.*

[*]Corresponding authors. E-mail: mh@nankai.edu.cn (Mei-Hui Yu); changze@nankai.edu.cn (Ze Chang).

Keywords: metal-organic frameworks, supramolecular linker, molecular pockets, ethylene purification, hydrocarbon separation

[#] J.-P. Han and H. Fang contributed equally to this work.

Table of Contents

S1. Materials and General Procedures 3

S2. Single Crystal X-Ray Crystallographic Data 9

S3. Nuclear Magnetic Resonance Spectrum 11

S4. Connolly Surface Analysis 11

S5. Additional Structural Figures 11

S6. Powder X-Ray Diffraction Patterns 12

S7. *In situ* variable-temperature PXRD Patterns 14

S8. Thermogravimetric Analysis 15

S9. BET Fitting Result 12

S10. *In situ* variable-pressure PXRD Patterns 14

S11. Low-pressure Gas Adsorption Isotherms 18

S12. Isosteric Heat of Adsorption (Q_{st}) Calculations 19

S13. Ideal Adsorption Solution Theory (IAST) Calculations 21

S14. Calculation of Separation Potential 22

S15. FTIR Spectrum 23

S16. Breakthrough experiments 22

S17. References 27

S1. Materials and General Procedures

All reagents and solvents were obtained from commercial sources and used without further purification. Among them, Zinc nitrate hexahydrate ($Zn(NO_3)_2$ $6H_2O$, CAS: 10196-18-6, purity 99%) was purchased from Aladdin Co. Ltd. 5,5'-di(1H-1,2,4-triazol-1-yl)-[1,1'-biphenyl]-3,3'-dicarboxylic acid (H_2DTBDC, CAS: 2097938-53-7, purity 98%) was purchased from Shanghai Tengqian Biotechnology Co. Ltd. N,N-Dimethylformamide (DMF, CAS: 68-12-2, purity 99.5%) was purchased from Tianjin Bohua Chemical Reagent Co. Ltd. Tetrafluoroboric acid (HBF_4, CAS: 14874-70-5, purity 40% in H_2O) was purchased from Meryer Co. Ltd.

Synthesis of NKM-47.

$Zn(NO_3)_2$ $6H_2O$ (0.1 mmol, 29.7 mg) and H_2DTBDC (0.02 mmol, 7.5 mg) were mixed and dissolved in 4 mL DMF and 80 μL HBF_4 in a 10 mL sealed glass vial. After 30 minutes of ultrasonic treatment, the glass vials were placed in an oven at 92 ℃ for 48 hours. After washing several times with DMF, colorless transparent fusiform crystals were collected in an overall yield of about 54% (based on H_2DTBDC). Selected FTIR (KBr, cm^{-1}): 3530(br), 3114(w), 1577(vs), 1385(s), 1351(s), 1277(m), 1220(m), 1149(m), 1083(m), 994(m), 881(m), 787(s), 748(s), 664(s), 536(vs). Elem. Anal. calcd. (%) for $C_{447}H_{643}N_{149}O_{229}Zn_{20}$: C, 41.06; H, 4.96; N, 15.96. Found: C, 41.03; H, 5.16; N, 16.04.

Single Crystal X-Ray Diffraction.

Single-crystal X-ray diffraction (SCXRD) data for **NKM-47** were obtained on a Rigaku XtaLAB Synergy-R (Cu Kα, λ = 1.5418 Å) at 100 K. Lorentz polarization and absorption corrections were applied. The crystal structure was solved by Direct Methods and refined on F^2 by full-matrix least squares using the SHELXT package contained in Olex2 [1,2]. Non-hydrogen atoms were refined with anisotropic displacement parameters during the final cycles. Hydrogen atoms were located at geometrically calculated positions relative to their carrier atoms and refined with isotropic thermal parameters included in the final stage of the refinement. The contributions of heavily disordered solvent molecules were treated as diffuse using the Squeeze procedure implemented in the PLATON program [3,4]. A summary of the crystallographic data is given in Table S2.

Powder X-Ray Diffraction and *In situ* variable-temperature PXRD.

Powder X-ray diffraction (PXRD) was performed on a Rigaku MiniFlex 600 diffractometer with 40 kV and 15 mA copper target tube and graphite monochromator. The corresponding scan speed and step size were 10 °/min and 0.02 °, respectively. The powder samples were made from ground crystals.

In situ variable-temperature PXRD data were collected in the range of 3 ° to 50 ° using a Rigaku MiniFlex 600 diffractometer in the Ar atmosphere. Anton Paar's BTS 500 stage was employed to regulate the test temperature and record variable-temperature PXRD data.

Nuclear Magnetic Resonance

Nuclear magnetic resonance (NMR) spectrum was recorded on Bruker Avance Neo 400MHz instrument and calibrated using residual undeuterated solvent (D_6-DMSO) as an internal reference.

***In situ* variable-pressure PXRD.**

In situ variable-pressure PXRD data were recorded on a Rigaku Smart Lab instrument operating at 40 kV and 40 mA, equipped with an Anton Paar TTK 600 accessory. The experimental setup included a combined control unit for temperature regulation, with the chamber connected to a turbo vacuum pump, gas injection lines, and pressure detectors to create a controlled environment for the samples. Prior to testing, the **NKM-47** samples underwent activation at 80 ℃ under vacuum.

For the experiments, **NKM-47** samples were loaded into the chamber, ensuring a flat sample surface. An air refrigerator cryo-system, coupled with the Temperature Control Unit in the Anton Paar TTK 600 stage, was connected to the sample chamber to regulate the temperature range. The sealed samples were vacuumed for 3 hours, then PXRD data were collected at 0 bar. Subsequently, the samples were loaded with N_2 at 20 mL min^{-1}, and pressure was monitored using pressure detectors. PXRD patterns were recorded after equilibration (1 % of pressure change within 10 min) at selected pressure points. *In situ* PXRD patterns were measured at each adsorption and desorption equilibrium point under controlled temperature and pressure conditions.

Thermogravimetric Analysis Tests.

Thermogravimetric analysis (TGA) was performed using a Rigaku TG8121 thermal analyzer under argon protection at a rate of 10 ℃/min from room temperature to 800 ℃, using an empty Al_2O_3 ceramic as a reference.

Gas Sorption Measurements.

The N_2 adsorption experiment was performed on a Micrometrics ASAP 2460 gas adsorption analyzer. Initially, the sample was soaked in fresh acetone, and the solvent was exchanged nine times for three days. Subsequently, a sufficient amount of target sample was placed in a sample tube and heated *in situ* at 80 ℃ for 12 h on a Micrometrics Smart VacPrep degassing station to remove solvent molecules, using more than 100 mg of desolvated sample per measurement. The N_2 adsorption isotherm of the activated sample was measured at 77 K. Light hydrocarbon adsorption testing was done on a Micrometrics 3Flex gas adsorption analyzer. After undergoing the solvent-exchanged process, the synthesized sample was degassed in sample tubes for 12 h at 80 ℃, and more than 100 mg of desolvated samples were used for adsorption measurements. Single-component gas adsorption isotherms of C_2H_2, C_2H_6, and C_2H_4 were performed at 273 K and 298 K, respectively.

Isosteric Heat of Adsorption (Q_{st}) Calculations

The dual-Langmuir equation was used to calculate the isosteric heat of adsorption for C_2H_2, C_2H_6, and C_2H_4 at 273 K and 298 K for **NKM-47**. At two temperatures, the data were fitted using the dual-Langmuir equation:

$$q = \frac{q_{A,sat} b_A P}{1 + b_A P} + \frac{q_{B,sat} b_B P}{1 + b_B P}$$

Here, P is the pressure (Pa), q is the adsorbed amount (mmol g^{-1}), $q_{A,sat}$ and $q_{B,sat}$ are the saturation capacities of sites A and B (mmol g^{-1}), b_A and b_B are the affinity coefficients of sites A and B (Pa^{-1}). Based on the dual-Langmuir coefficients obtained from the fitted isotherms, the values of α, β and p corresponding to the specified temperatures can be sequentially obtained using the following three formulas：

$$\alpha = \left(q_{A,sat} + q_{B,sat} - q\right) b_A b_B$$

$$\beta = \left(q_{A,sat} - q\right) b_A + \left(q_{B,sat} - q\right) b_B$$

$$p = \frac{\sqrt{\beta^2 + 4\alpha q} - \beta}{2\alpha}$$

Finally, the isosteric heat of adsorption (Q_{st}) was calculated using the following equation:

$$Q_{st} = (\ln p_1 - \ln p_2) \times \frac{R T_1 T_2}{T_1 - T_2}$$

Q_{st} is the coverage-dependent isosteric heat of adsorption (kJ mmol^{-1}), R is the universal gas constant (8.314 J mol^{-1} K^{-1}), T_1 and T_2 represent the two temperatures at which the adsorption

isotherm measurements are conducted (K), p_1 and p_2 are the pressure points corresponding to the temperatures T_1 and T_2 (Pa), respectively.

Ideal Adsorption Solution Theory (IAST) Calculations

The Ideal Adsorbed Solution Theory (IAST) is used to predict binary gas mixtures' adsorption selectivity based on single-component isotherms. Among various models, the dual-Langmuir-Freundlich model is the most suitable for fitting pure component adsorption isotherms. The dual-Langmuir-Freundlich model is defined by:

$$q = \frac{q_{A,sat} b_A P^{1/n_1}}{1 + b_A P^{1/n_1}} + \frac{q_{B,sat} b_B P^{1/n_2}}{1 + b_B P^{1/n_2}}$$

Herein, P is the pressure (kPa), q is the adsorbed amount (mmol g^{-1}), $q_{A,sat}$ and $q_{B,sat}$ are the saturation capacities of sites A and B (mmol g^{-1}), b_A and b_B are the affinity coefficients of sites A and B (kPa^{-1}), and n_1 and n_2 represent the deviations from an ideal homogeneous surface. The fitted parameters were used to predict multi-component adsorption with IAST.

The selectivity S_{ads} of component A for preferential adsorption over component B in a binary mixture is defined as:

$$S_{ads} = \frac{q_1/q_2}{p_1/p_2}$$

In this case, q_1 and q_2 are the molar loadings of the adsorbed phase at partial pressures p_1 and p_2, respectively, in equilibrium with the bulk gas phase.

Calculations of Separation Potential

The separation potential (Δq) is a comprehensive parameter that integrates uptake capacity and selectivity, enabling the quantification of mixture separation in fixed bed adsorbers. The separation potential was calculated using the following equation:

$$\Delta q = q_1 \frac{y_2}{y_1} - q_2$$

Wherein, the q_1 and q_2 represent the molar loadings within the MOF that is in equilibrium with a bulk gas phase mixture with mole fractions y_1, and $y_2 = 1 - y_1$.

Theoretical calculations

DFT calculations were carried out using the CP2K code [5]. All calculations employed a mixed Gaussian and planewave basis sets. Core electrons were represented with norm-conserving Goedecker-Teter-Hutter pseudopotentials [6-8], and the valence electron wavefunction was expanded in a double-zeta basis set with polarization functions along with

an auxiliary plane wave basis set with an energy cutoff of 360 Ry [9]. The generalized gradient approximation exchange-correlation functional of Perdew, Burke, and Ernzerhof (PBE) was used [10]. Each configuration was optimized with the Broyden-Fletcher-Goldfarb-Shanno (BFGS) algorithm with SCF convergence criteria of 1.0×10^{-8} au. To compensate for the long-range van der Waals dispersion interaction between the adsorbate and the MOF, the DFT-D3 scheme with an empirical damped potential term was added into the energies obtained from the exchange-correlation functional in all calculations [11].

The adsorption energy between the adsorbate and the **NKM-47** substrate can be calculated using the following equation:

$$\Delta E_{ads} = E_{adsorbate@substrate} - E_{substrate} - E_{adsorbate}$$

In the equation, $E_{adsorbate@substrate}$ and $E_{substrate}$ represent the total energies of the substrate with and without adsorbate, respectively. $E_{adsorbate}$ is the total energy of the adsorbate. According to this equation, a negative adsorption energy corresponds to a stable adsorption structure.

Breakthrough experiments.

The breakthrough experiments used the BEISHIDE multi-constituent adsorption breakthrough curve analyzer. The flow rates of all gases were regulated using mass flow controllers, while a mass spectrometer monitored the exhaust gas from the column. Mixed gases of C_2H_2/C_2H_4 (10/90, v/v), C_2H_6/C_2H_4 (10/90, v/v), and $C_2H_2/C_2H_6/C_2H_4$ (10/10/80, v/v) were introduced into the breakthrough apparatus at a rate of 4.0 mL min^{-1} at 298 K and 1 bar. For the $C_2H_2/C_2H_6/C_2H_4$ (1/9/90, v/v/v) with the rate of 20.0 mL min^{-1} at 298 K and 1 bar, gas monitoring within the column was performed using an online infrared detection system.

***In situ* infrared (IR) measurements.**

In situ fourier transform infrared (FTIR) measurements were performed on a TENSOR 37 FT-IR spectrometer. Grind a small amount of sample (10 mg) with KBr and press into a transparent disc by using a tablet press. Before measurement, the sample was activated at 353 K under a purging N_2 gas with a flow of 50 mL min^{-1} for 2 h, and then cooled back to room temperature for C_2H_2, C_2H_6 or C_2H_4 exposure measurement. The gas flow of C_2H_2, C_2H_6 or C_2H_4 was introduced with the rate of 1 mL min^{-1} and the IR spectra were recorded at different times.

***In situ* Raman measurements.**

In situ Raman measurements were performed on RMS1000 with a 785 nm laser excitation. Before measurement, the sample chamber was evacuated to vacuum, and the Raman spectrum of the sample was acquired at 0 bar. Subsequently, exposure measurements were conducted separately with C_2H_2, C_2H_6 or C_2H_4 by introducing a flow of the respective gas until a pressure of 1 bar was attained, while recording the Raman spectra.

Table S1. Physicochemical Properties of C_2H_2, C_2H_4, and C_2H_6.

	Boiling point (K)	Molecular dimensions (Å^3)	Kinetic diameter (Å)	Polarizability ×10^{-25} (cm^{-3})	Quadrupole Moment (×10^{26}/esu cm^2)
C_2H_2	188.4	3.32×3.34×5.70	3.33	33.3—33.9	+7.2
C_2H_4	169.4	3.28×4.18×4.84	4.16	42.5	+1.5
C_2H_6	184.6	3.81×4.08×4.82	4.44	44.3—44.7	+0.65

S2. Single Crystal X-Ray Crystallographic Data

Table S2. Crystal data and structure refinement for **NKM-47**.

Identification code	**NKM-47**
CCDC number	2452601
Empirical formula	$C_{36}H_{20}N_{12}O_8Zn_2$
Formula weight	879.38
Temperature/K	100.00(10)
Crystal system	monoclinic
Space group	$C2/c$
a/Å	13.43600(10)
b/Å	36.2214(3)
c/Å	23.0313(2)
α/ °	90
β/ °	92.0450(10)
γ/ °	90
Volume/Å^3	11201.52(16)
Z	8
ρ_{calc}g/cm^3	1.043
μ/mm^{-1}	1.452
F(000)	3552.0
Crystal size/mm^3	0.08 × 0.04 × 0.03
Radiation	Cu Kα (λ = 1.54184 Å)
2θ range for data collection/ °	8.116 to 152.008
Index ranges	$-13 \leq h \leq 16$, $-42 \leq k \leq 45$, $-28 \leq l \leq 28$
Reflections collected	27036
Independent reflections	11211 [R_{int} = 0.0217, R_{sigma} = 0.0242]
Data/restraints/parameters	11211/0/523
Goodness-of-fit on F^2	1.092
Final R indexes [I>=2σ (I)]	$R1$ = 0.0463, $wR2$ = 0.1371
Final R indexes [all data]	$R1$ = 0.0494, $wR2$ = 0.1394
Largest diff. peak/hole / e Å^{-3}	1.29/−0.55

S3. Nuclear Magnetic Resonance Spectrum

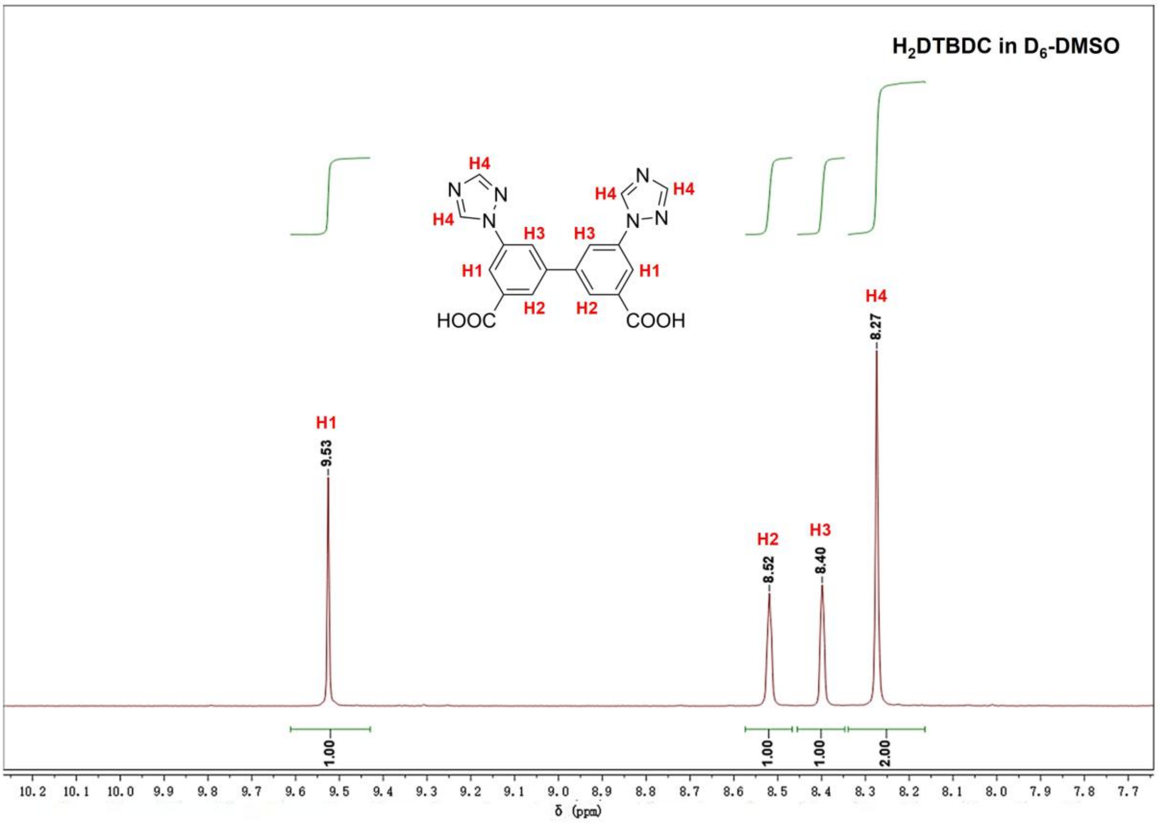

Figure S1. ^{1}H NMR spectrum of H_2DTBDC in D_6-DMSO.

S4. Connolly Surface Analysis

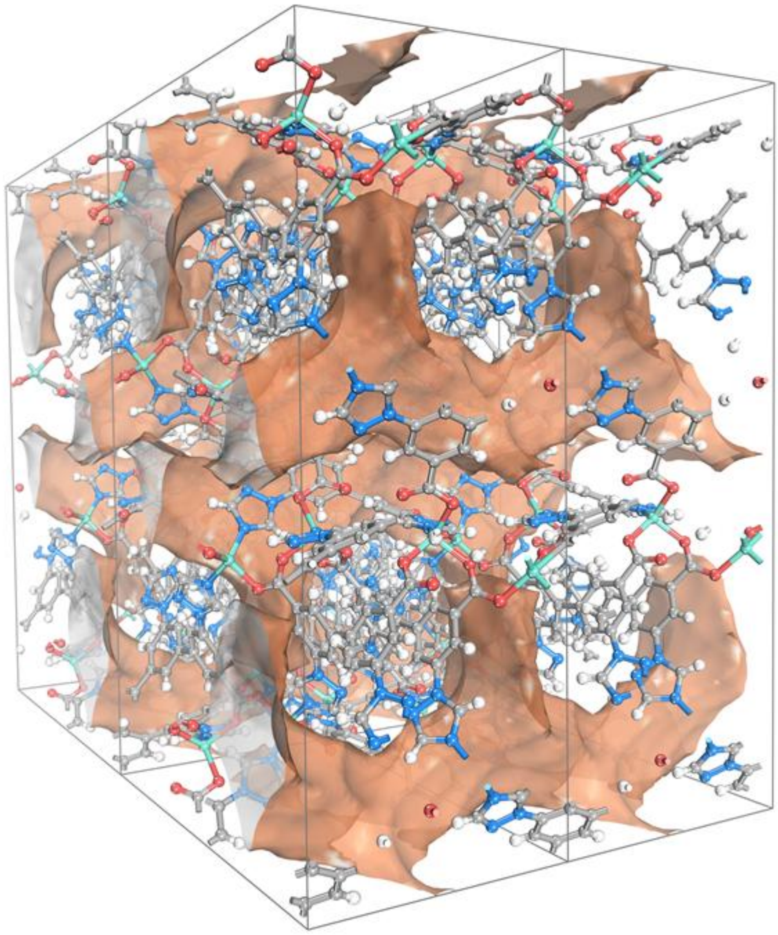

Figure S2. Connolly surface representation of **NKM-47**, illustrating the channels for gas molecules diffusion and the pocket-like adsorption sites for gas molecules accommodation.

S5. Additional Structural Figures

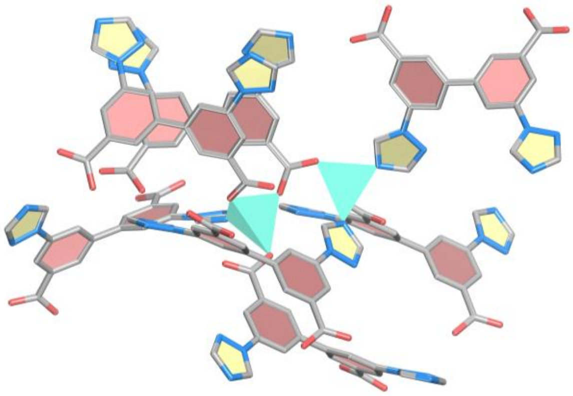

Figure S3. The coordination mode of the molecular building blocks.

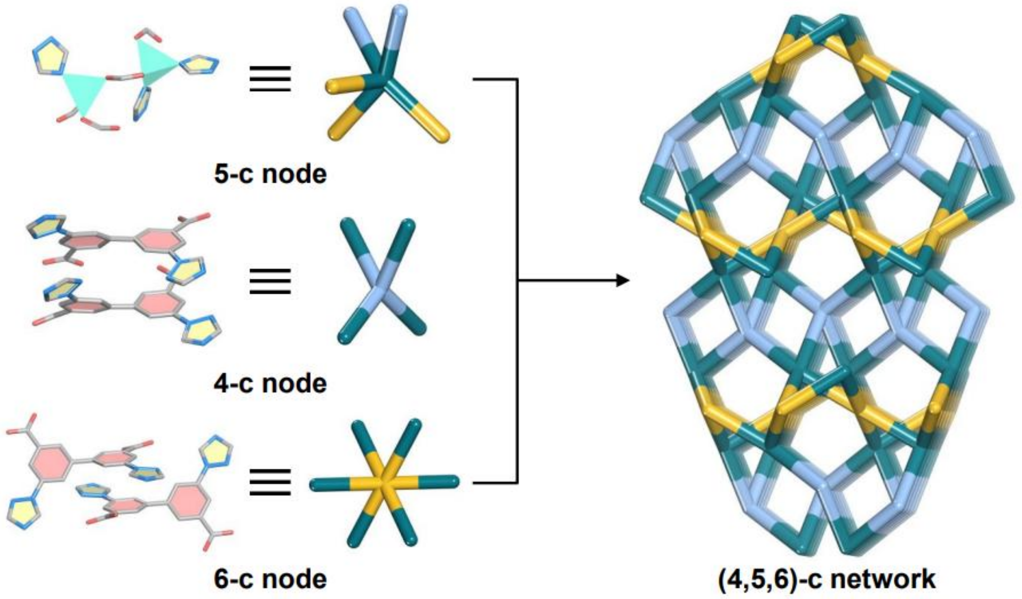

Figure S4. The topology analysis of **NKM-47**.

S6. Powder X-Ray Diffraction Patterns

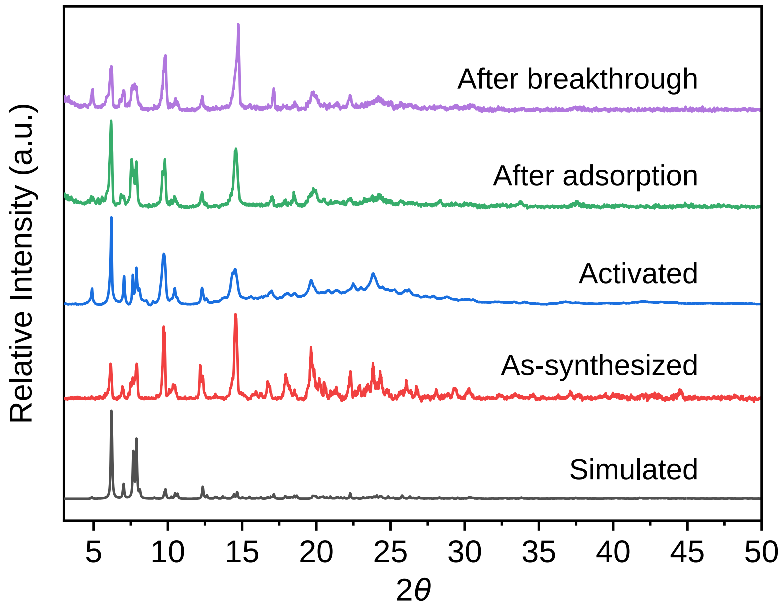

Figure S5. PXRD patterns for **NKM-47**, including the simulated pattern, as-synthesized sample, activated sample, and samples after adsorption and breakthrough measurements.

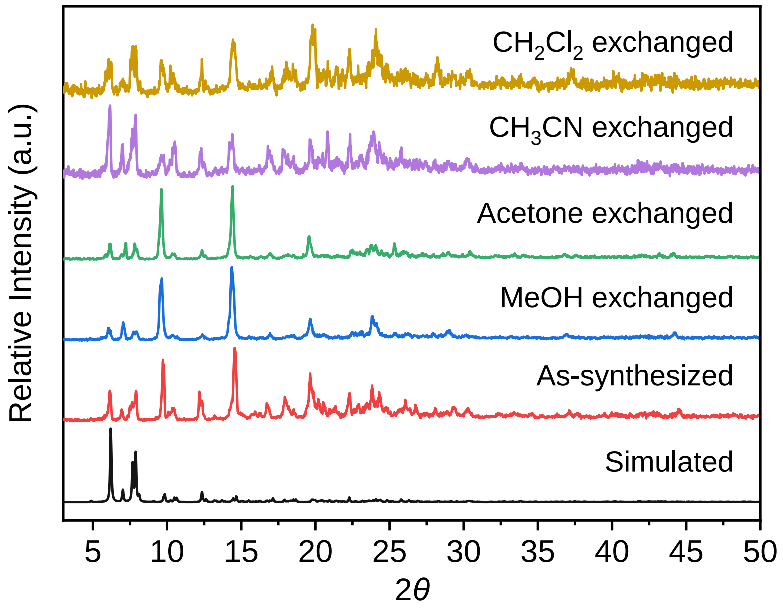

Figure S6. PXRD patterns for **NKM-47** soaked in different solvents for 3 days.

S7. *In situ* variable-temperature PXRD Patterns

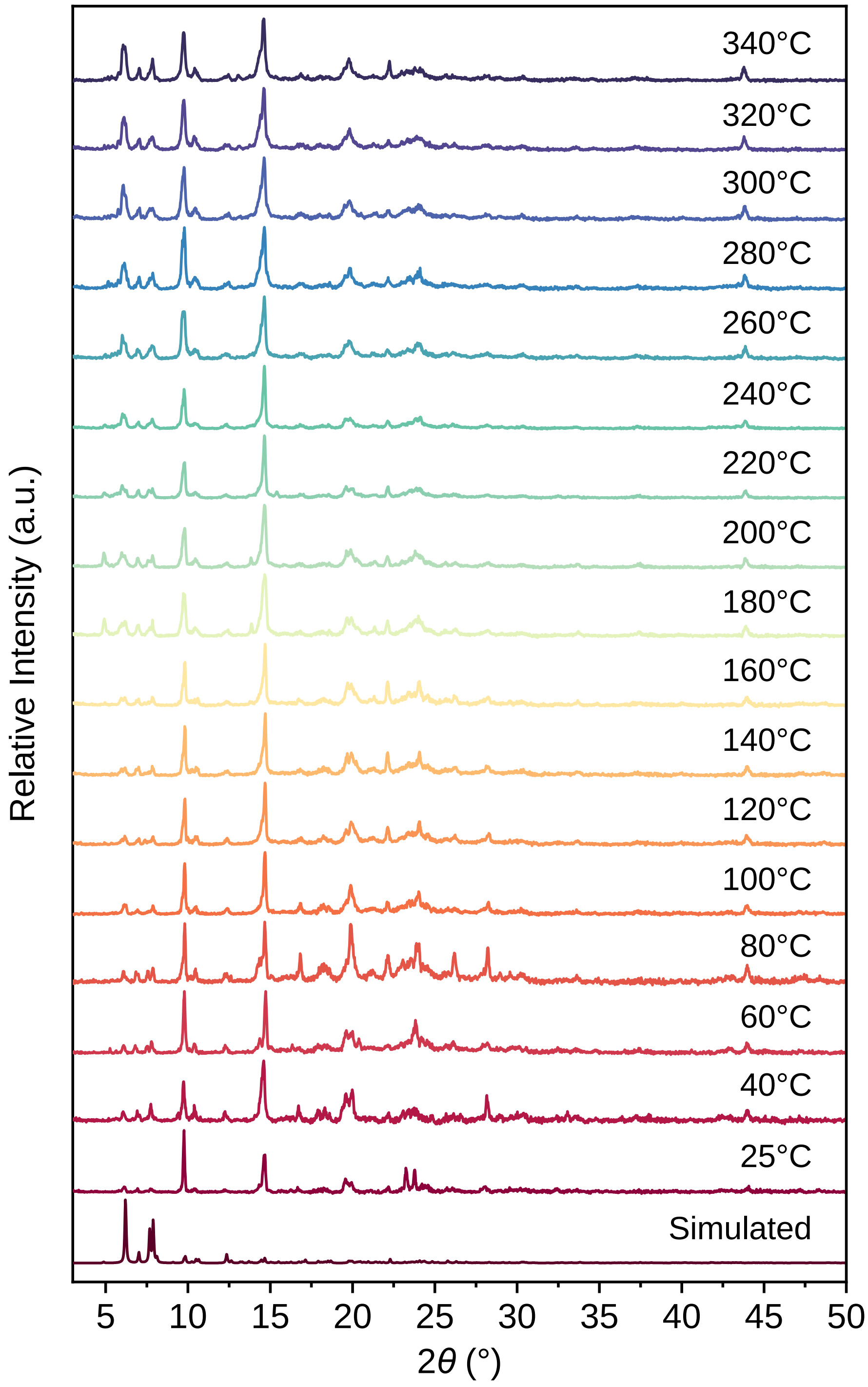

Figure S7. The variable-temperature PXRD patterns of **NKM-47**.

S8. Thermogravimetric Analysis

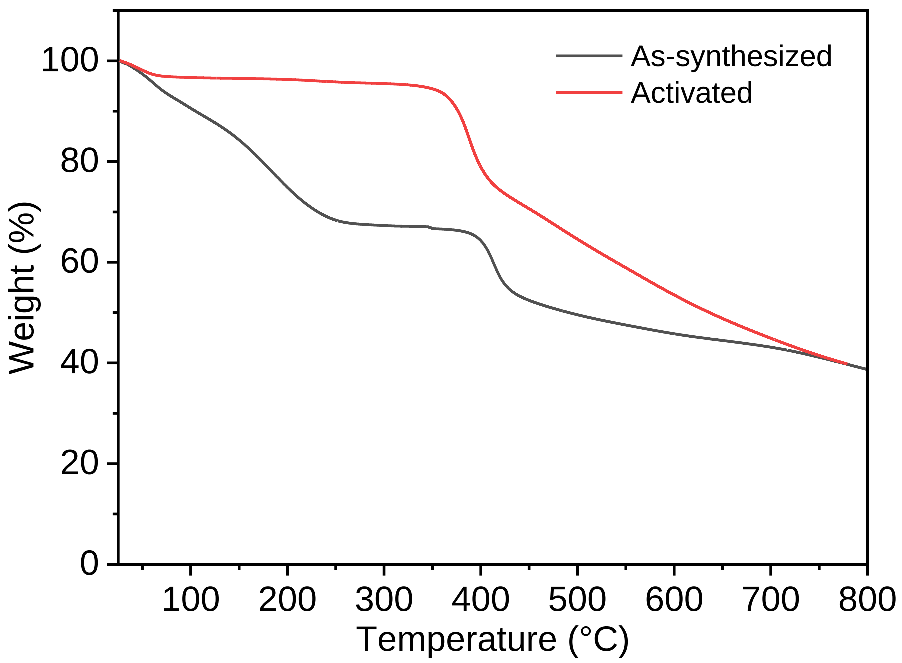

Figure S8. Thermogravimetric analysis of the as-synthesized and activated **NKM-47**.

S9. BET Fitting Result

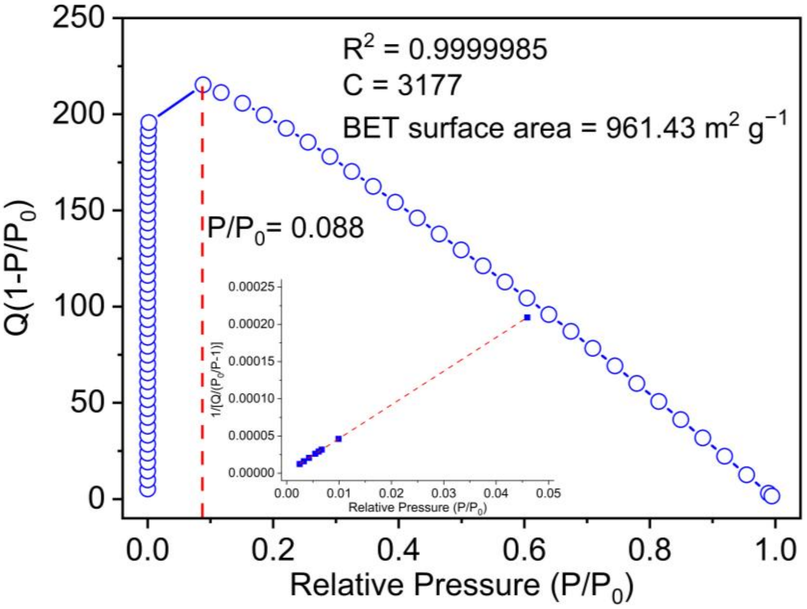

Figure S9. Q(1–P/P_0) vs. P/P_0 for **NKM-47** isotherm, only the range below P/P_0 = 0.088 satisfies the first consistency criterion for applying the BET theory; inset: plot of the linear region for the BET equation.

S10. *In situ* variable-pressure PXRD Patterns

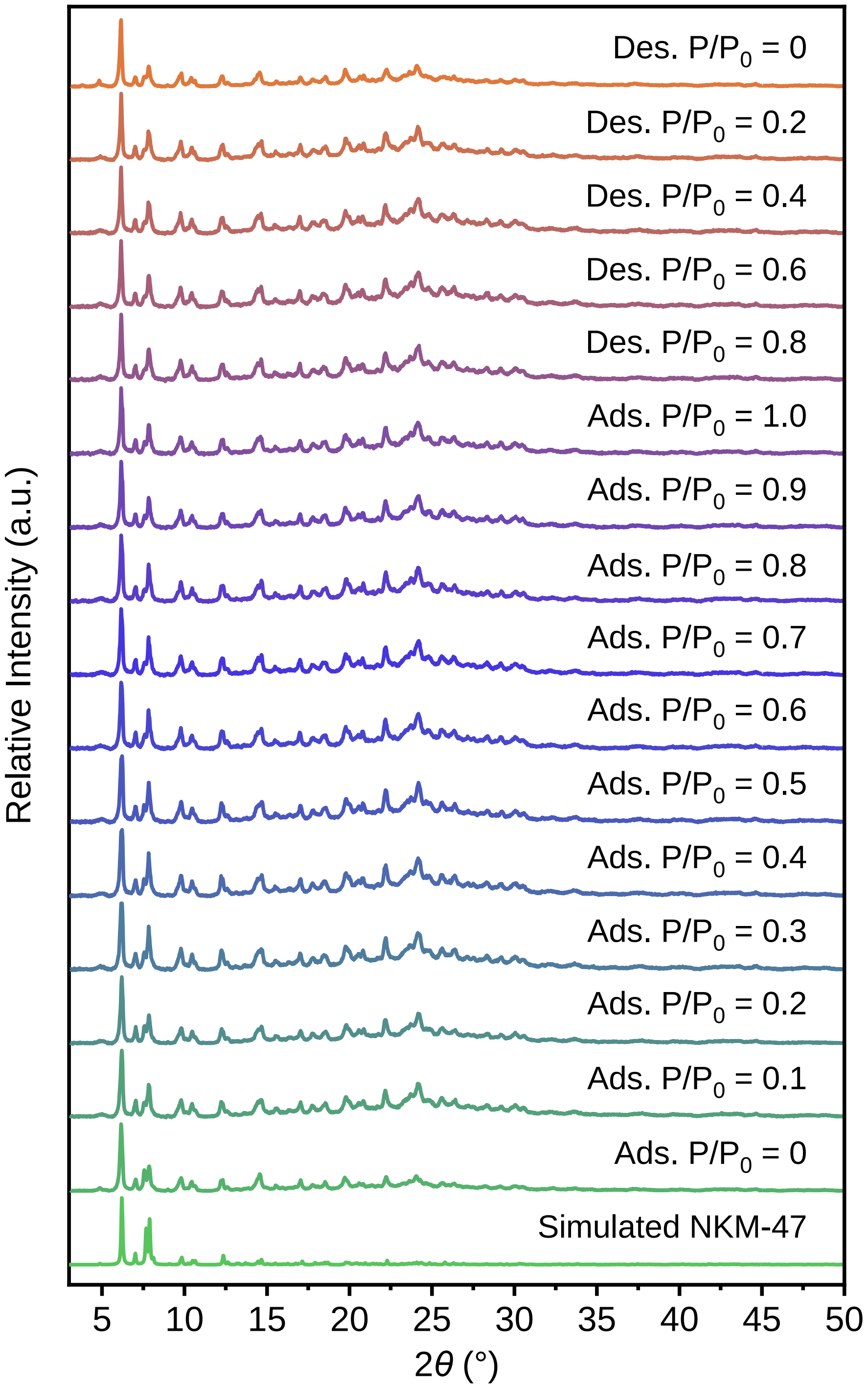

Figure S10. *In situ* PXRD of **NKM-47** during the adsorption and desorption of N_2 at 77 K.

S11. Low-pressure Gas Adsorption Isotherms

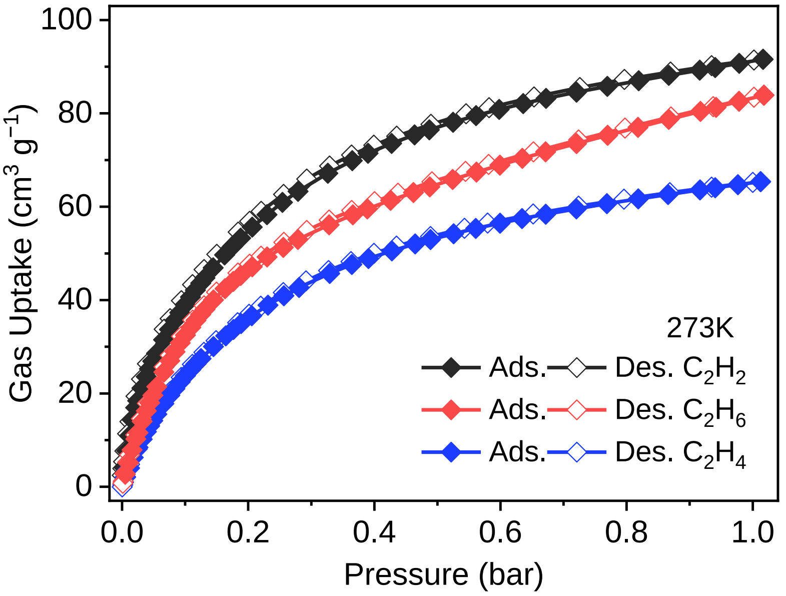

Figure S11. C_2H_2, C_2H_6, and C_2H_4 adsorption isotherms measured at 273 K for **NKM-47**.

S12. Isosteric Heat of Adsorption (Q_{st}) Calculations

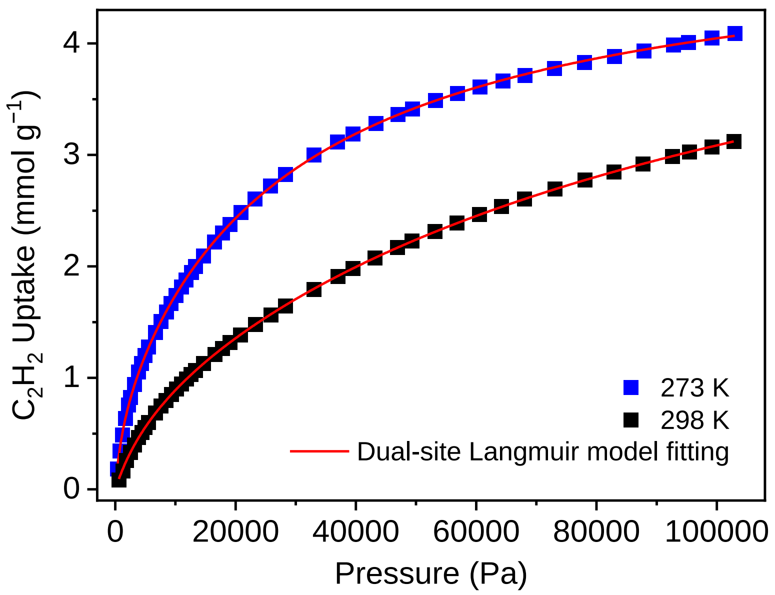

Figure S12. The details of the dual-site Langmuir equation (solid lines) fitting to the experimental C_2H_2 adsorption data (symbols) for **NKM-47**.

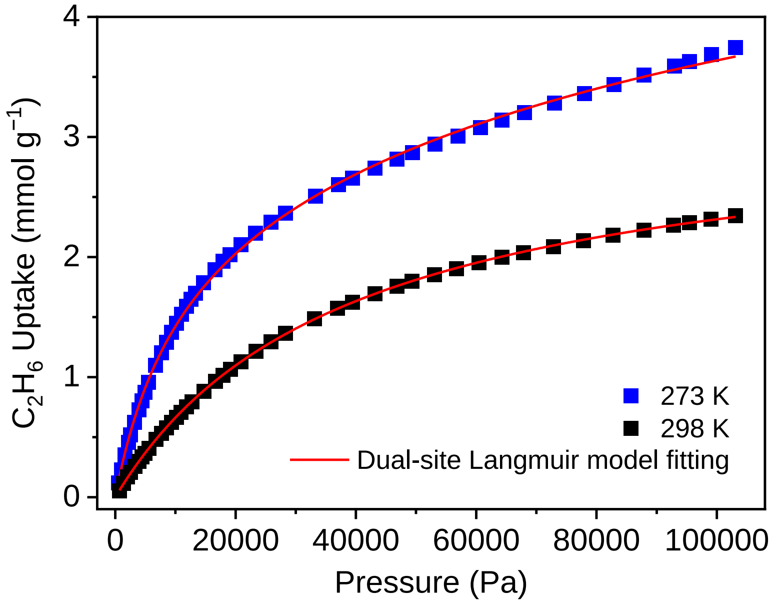

Figure S13. The details of the dual-site Langmuir equation (solid lines) fitting to the experimental C_2H_6 adsorption data (symbols) for **NKM-47**.

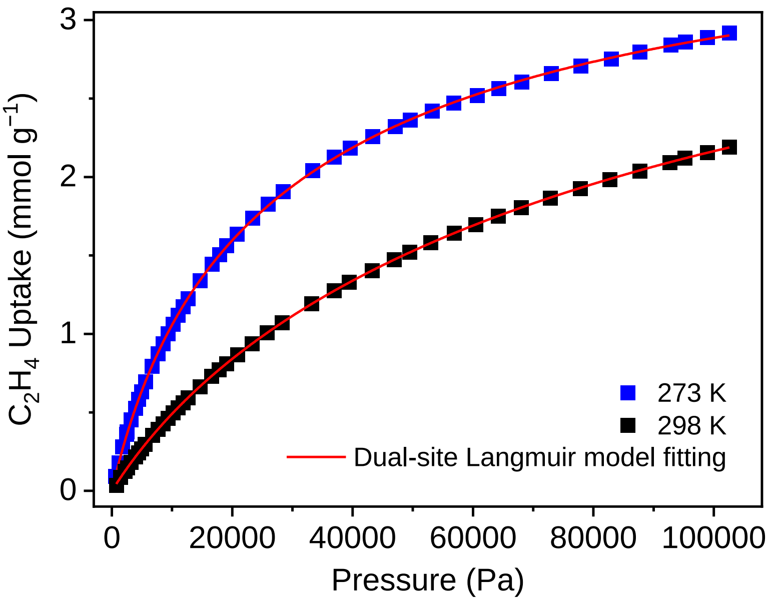

Figure S14. The details of the dual-site Langmuir equation (solid lines) fitting to the experimental C_2H_4 adsorption data (symbols) for **NKM-47**.

Table S3. The obtained dual-site Langmuir model fitting parameters for **NKM-47**.

MOF	**NKM-47**					
Gas	C_2H_2		C_2H_6		C_2H_4	
Temperature	273 K	298 K	273 K	298 K	273 K	298 K
b_A	7.0172E−4	1.54133E−4	9.96491E−5	2.77829E−5	7.83872E−5	4.66207E−5
b_B	3.41547E−5	9.21458E−6	7.24732E−6	1.79736E−5	2.14429E−5	9.71009E−6
$q_{A,sat}$	0.74185	0.78608	2.37472	2.64742	1.47728	0.59923
$q_{B,sat}$	4.28492	4.89529	3.52104	0.57225	2.31142	3.39318
R^2	0.99993	0.99999	0.99921	0.99996	0.99994	0.99999

S13. Ideal Adsorption Solution Theory (IAST) Calculations

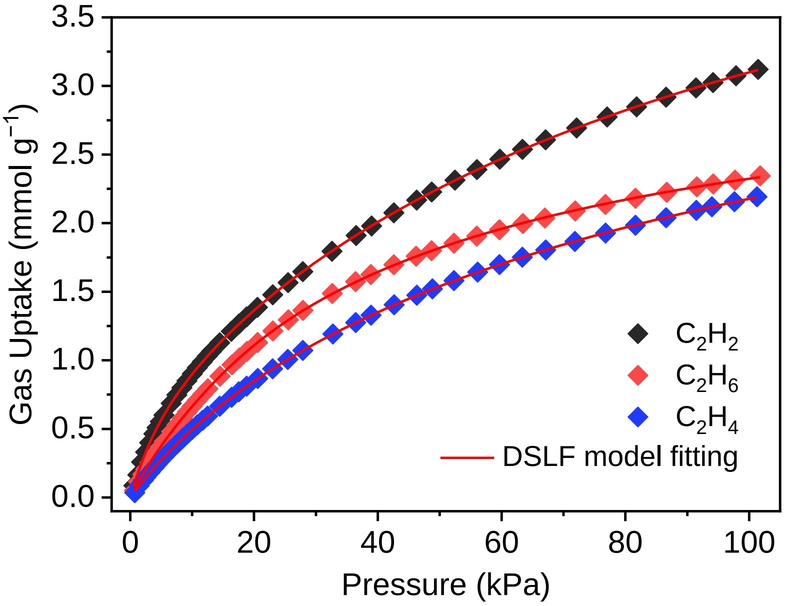

Figure S15. Dual-site Langmuir–Freundlich (DSLF) equation fits (lines) of C_2H_2, C_2H_6, and C_2H_4 adsorption isotherms (points) measured at 298 K for **NKM-47**.

Table S4. The obtained dual-site Langmuir–Freundlich (DSLF) model fitting parameters for **NKM-47**.

MOF	**NKM-47**		
Gas	C_2H_2	C_2H_6	C_2H_4
$q_{A,sat}$	0.76731	3.25871	0.73831
$q_{B,sat}$	4.54976	0.0711	3.29754
b_A	0.16236	0.02808	0.0416
b_B	0.00805	0.0000724315	0.00949
n_1	1.08931	0.95103	0.98845
n_2	1.06375	3.36496	0.99547
R^2	0.99999	0.99998	0.99999

S14. Calculation of Separation Potential

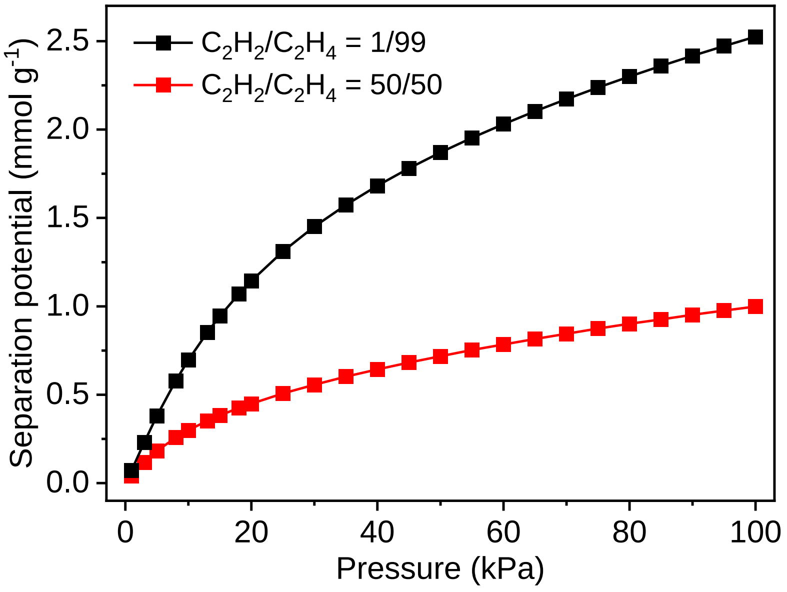

Figure S16. Comparisons of separation potential for C_2H_2/C_2H_4 (1/99, 50/50) mixtures of **NKM-47**.

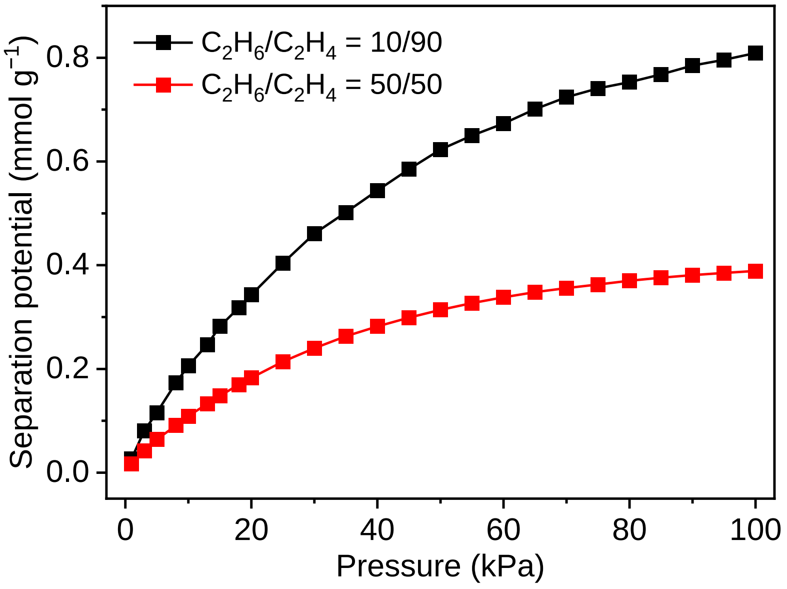

Figure S17. Comparisons of separation potential for C_2H_6/C_2H_4 (10/90, 50/50) mixtures of **NKM-47**.

S15. FTIR Spectrum

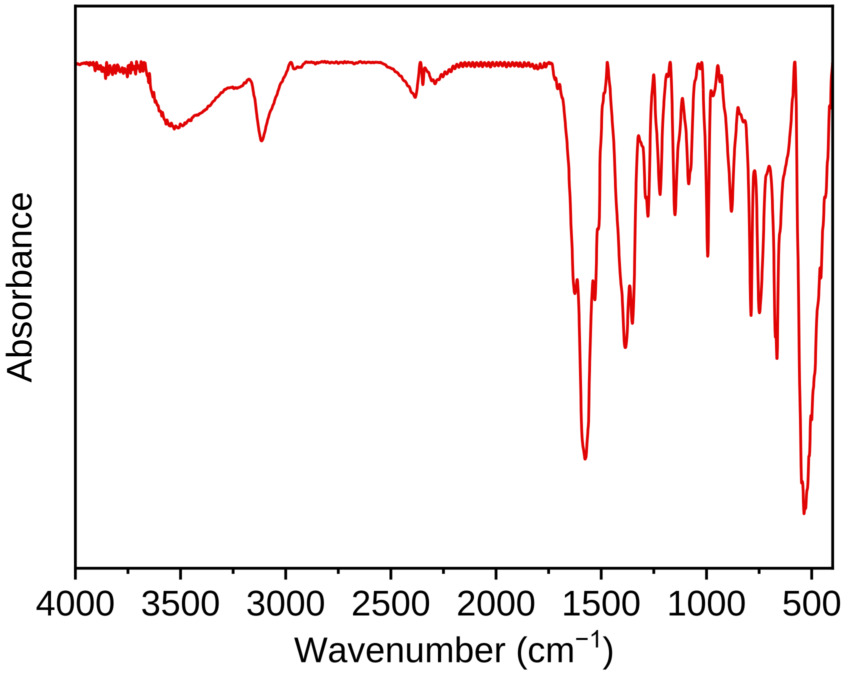

Figure S18. FTIR spectrum of the activated **NKM-47**.

S16. Breakthrough experiments

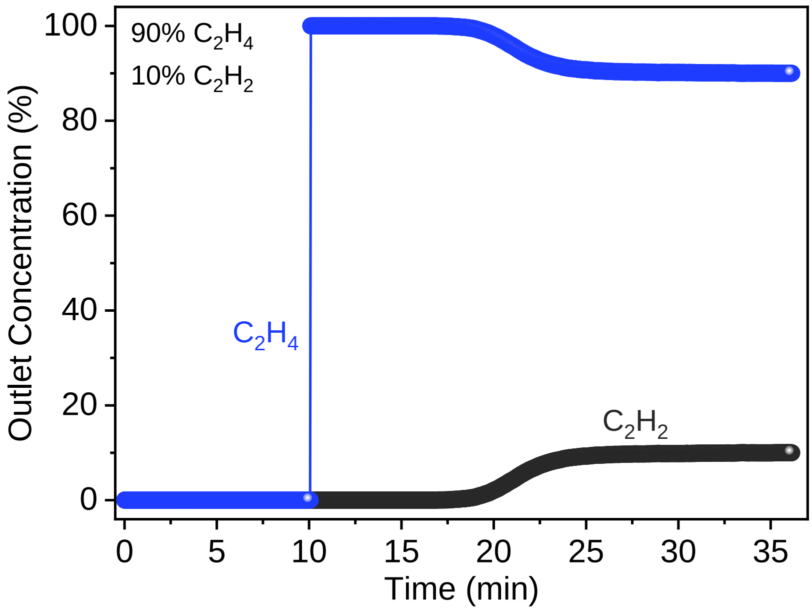

Figure S19. Experimental breakthrough curves of **NKM-47** for 10/90 C_2H_2/C_2H_4 mixtures with a flow rate of 4.0 mL min^{-1} at 298 K and 1 bar.

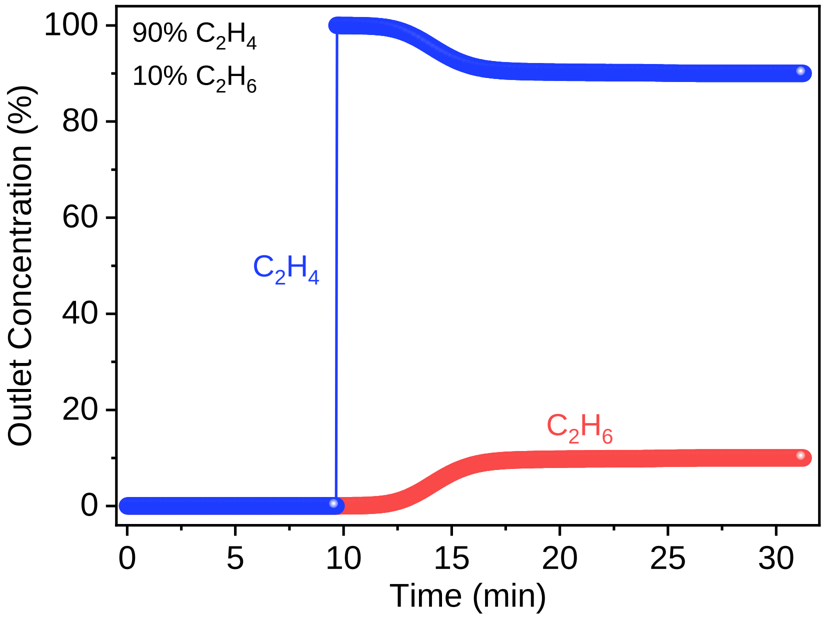

Figure S20. Experimental breakthrough curves of **NKM-47** for 10/90 C_2H_6/C_2H_4 mixtures with a flow rate of 4.0 mL min^{-1} at 298 K and 1 bar.

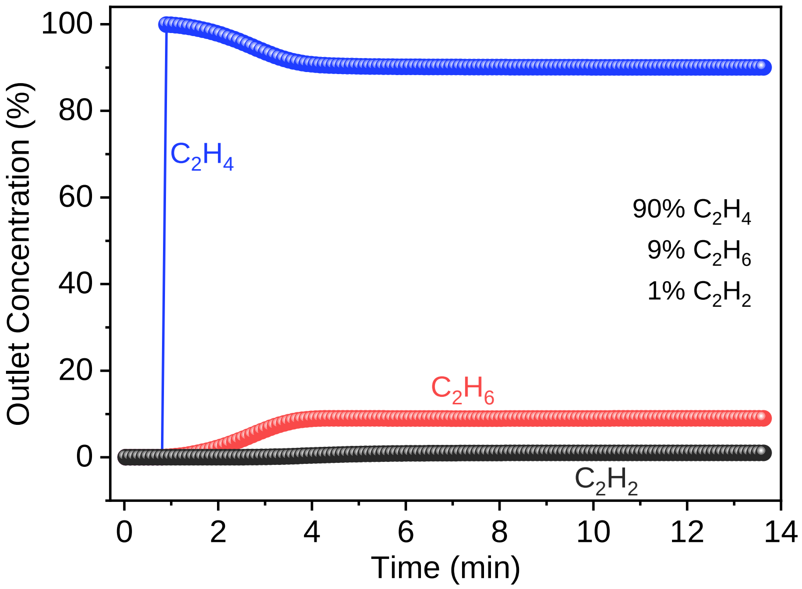

Figure S21. Experimental breakthrough curves of **NKM-47** for 1/9/90 $C_2H_2/C_2H_6/C_2H_4$ ternary mixtures with a flow rate of 20.0 mL min^{-1} at 298 K and 1 bar.

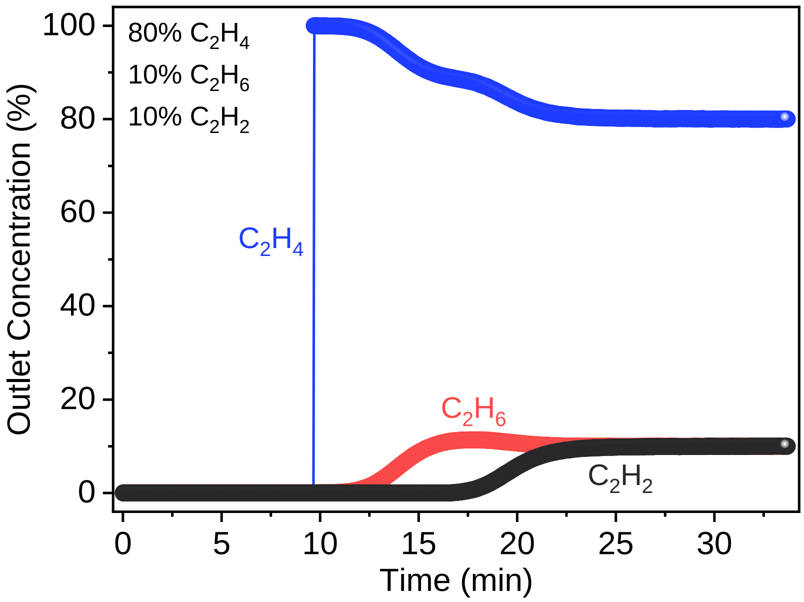

Figure S22. Experimental breakthrough curves of **NKM-47** for 10/10/80 $C_2H_2/C_2H_6/C_2H_4$ ternary mixtures with a flow rate of 4.0 mL min^{-1} at 298 K and 1 bar.

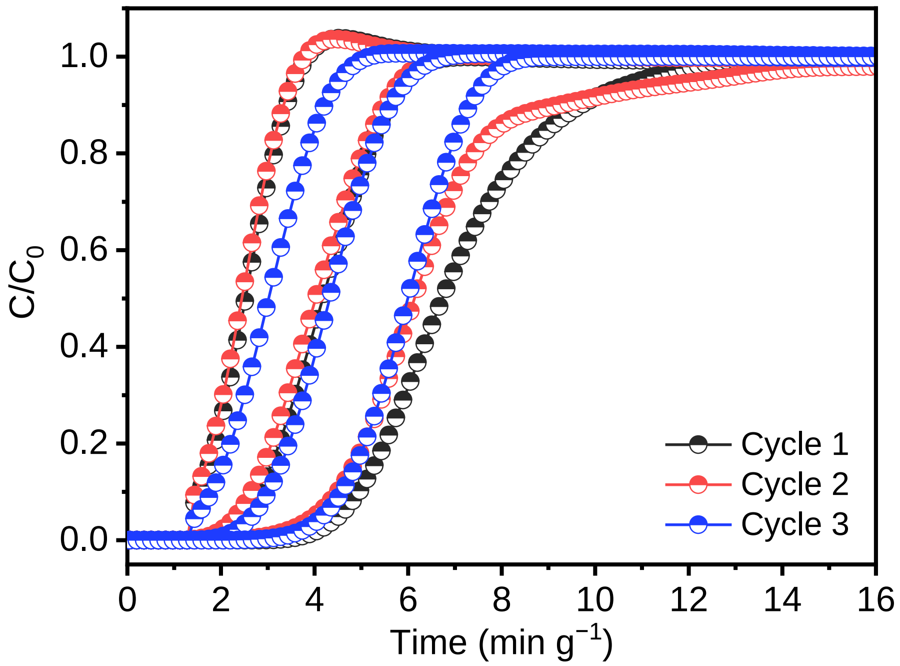

Figure S23. The cycling breakthrough experiments of 1/9/90 $C_2H_2/C_2H_6/C_2H_4$ mixtures under ambient conditions.

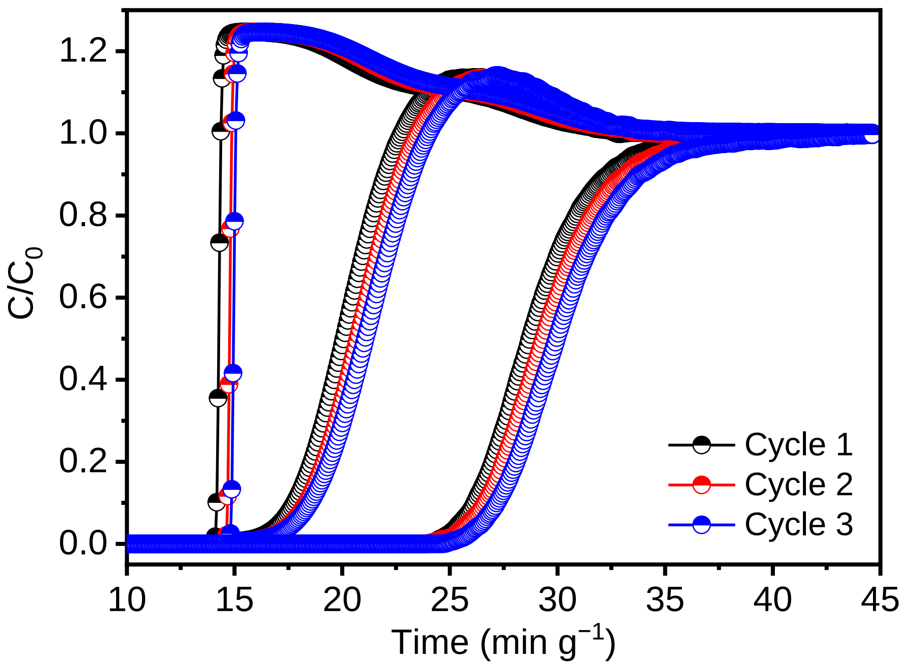

Figure S24. The cycling breakthrough experiments of 10/10/80 $C_2H_2/C_2H_6/C_2H_4$ mixtures under ambient conditions.

S17. References

1. Dolomanov OV, Bourhis LJ, Gildea RJ *et al.* OLEX2: a complete structure solution, refinement and analysis program. *J Appl Cryst* 2009; **42**: 339-41.
2. Sheldrick GM. SHELXT – Integrated space-group and crystal-structure determination. *Acta Cryst A* 2015; **71**: 3-8.
3. Van Der Sluis P, Spek AL. BYPASS: an effective method for the refinement of crystal structures containing disordered solvent regions. *Acta Cryst A* 1990; **46**: 194-201.
4. Spek AL. Single-crystal structure validation with the program PLATON. *J Appl Cryst* 2003; **36**: 7-13.
5. VandeVondele J, Krack M, Mohamed F *et al.* Quickstep: Fast and accurate density functional calculations using a mixed Gaussian and plane waves approach. *Comput Phys Commun* 2005; **167**: 103-28.
6. Hartwigsen C, Goedecker S, Hutter J. Relativistic separable dual-space Gaussian pseudopotentials from H to Rn. *Phys Rev B* 1998; **58**: 3641-62.
7. Goedecker S, Teter M, Hutter J. Separable dual-space Gaussian pseudopotentials. *Phys Rev B* 1996; **54**: 1703-10.
8. Krack M, Parrinello M. All-electron ab-initio molecular dynamics. *Phys Chem Chem Phys* 2000; **2**: 2105-12.
9. VandeVondele J, Hutter J. Gaussian basis sets for accurate calculations on molecular systems in gas and condensed phases. *J Chem Phys* 2007; **127**: 114105.
10. Perdew JP, Burke K, Ernzerhof M. Generalized Gradient Approximation Made Simple. *Phys Rev Lett* 1996; **77**: 3865-8.
11. Grimme S, Antony J, Ehrlich S *et al.* A consistent and accurate ab initio parametrization of density functional dispersion correction (DFT-D) for the 94 elements H-Pu. *J Chem Phys* 2010; **132**: 154104.
